# Effective Prevention and Treatment of Acute Leukemias in Mice by Activation of Thermogenic Adipose Tissues

**DOI:** 10.1002/advs.202402332

**Published:** 2024-07-25

**Authors:** Ruibo Chen, Tianran Cheng, Sisi Xie, Xiaoting Sun, Mingjia Chen, Shumin Zhao, Qingyan Ruan, Xiaolei Ni, Mei Rao, Xinyi Quan, Kaiwen Chen, Shiyue Zhang, Tao Cheng, Yuanfu Xu, Yuguo Chen, Yunlong Yang, Yihai Cao

**Affiliations:** ^1^ Department of Cellular and Genetic Medicine School of Basic Medical Sciences Fudan University Shanghai 200032 China; ^2^ State Key Laboratory of Experimental Hematology National Clinical Research Center for Blood Diseases Haihe Laboratory of Cell Ecosystem Institute of Hematology & Blood Diseases Hospital Chinese Academy of Medical Sciences & Peking Union Medical College Tianjin 300020 China; ^3^ Department of Cardiology Basic Scientific Research Center Longyan First Hospital Affiliated to Fujian Medical University Longyan 364000 China; ^4^ Oujiang Laboratory (Zhejiang Lab for Regenerative Medicine, Vision and Brain Health) School of Pharmaceutical Science Wenzhou Medical University Wenzhou 325035 China; ^5^ Department of Emergency Medicine Shandong Provincial Clinical Research Center for Emergency and Critical Care Medicine Medical and Pharmaceutical Basic Research Innovation Center of Emergency and Critical Care Medicine China’s Ministry of Education NMPA Key Laboratory for Clinical Research and Evaluation of Innovative Drug Shandong International Cooperative Laboratory for Emergency and Critical Care Medicine Qilu Hospital of Shandong University Jinan 250012 China; ^6^ Department of Microbiology Tumor and Cell Biology Karolinska Institutet Solna 17165 Sweden

**Keywords:** cancer therapy, glycolysis, leukemia, metabolism, thermogenic adipose tissue

## Abstract

Acute myeloid leukemia (AML) and acute lymphoblastic leukemia (ALL) are common hematological malignancies in adults. Despite considerable research advances, the development of standard therapies, supportive care, and prognosis for the majority of AML and ALL patients remains poor and the development of new effective therapy is urgently needed. Here, it is reported that activation of thermogenic adipose tissues (TATs) by cold exposure or β3‐adrenergic receptor agonists markedly alleviated the development and progression of AML and ALL in mouse leukemia models. TAT activation (TATA) monotherapy substantially reduces leukemic cells in bone marrow and peripheral blood, and suppresses leukemic cell invasion, including hepatomegaly and splenomegaly. Notably, TATA therapy prolongs the survivals of AML‐ and ALL‐bearing mice. Surgical removal of thermogenic brown adipose tissue (BAT) or genetic deletion of uncoupling protein 1 (UCP1) largely abolishes the TATA‐mediated anti‐leukemia effects. Metabolomic pathway analysis demonstrates that glycolytic metabolism, which is essential for anabolic leukemic cell growth, is severely impaired in TATA‐treated leukemic cells. Moreover, a combination of TATA therapy with chemotherapy produces enhanced anti‐leukemic effects and reduces chemotoxicity. These data provide a new TATA‐based therapeutic paradigm for the effective treatment of AML, ALL, and likely other types of hematological malignancies.

## Graphical abstract

Cold exposure induces the activation of thermogenic adipose tissues with significant glucose uptake and thermogenesis in mammals. Consequently, leukemia progression under cold exposure is inhibited through global glucose redistribution. Combining cold exposure with low‐dose chemotherapy has a robust anti‐leukemia effect and low toxicity, which is beneficial to patients with leukemia.

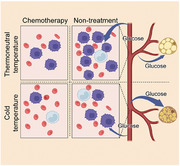

## 1 Introduction

Acute leukemias are common cancer types that affect all age groups around the globe.^[^ ^1^ ^]^ They constitute heterogeneous malignancies embroiling diverse genetic and environmental factors, with a higher prevalence in developed countries and a relatively higher mortality in developing countries.^[^ ^1^ ^]^ Acute leukemias arise from clonal lineages of malignant immature and precursor hematopoietic cells that are often located in bone marrow (BM).^[^ ^2^ ^]^

Acute myeloid leukemia (AML), originating from the cancerous myeloid lineage of hematopoietic cells, is the most common type of acute leukemia in adults.^[^ ^3^ ^]^ AML is characterized by the rapid growth of undifferentiated myeloid blasts. Despite improved survival and sometimes curative therapy in a small population of patients, the overall prognosis of AML is poor. Conventional chemotherapies such as anthracycline and cytarabine have been used for decades.^[^ ^4^ ^]^ Recent advances in defining molecular targets have provided new therapeutic options, particularly personalized therapeutic regimens for AML.^[^ ^5^ ^]^

Acute lymphoblastic leukemia (ALL) is the second most common acute leukemia in adults, with ≈75% of clinical cases originating from B‐lymphocyte precursors and 25% of cases from malignant T‐cell precursors.^[^ ^6^ ^]^ Diverse regimens, including vincristine, anthracycline, corticosteroids, and allogeneic stem cell transplantation, constitute the backbone of effective therapy.^[^ ^6^, ^7^ ^]^ However, only ≈30–40% of ALL patients achieve long‐term remission after treatment and elderly patients emanate poor prognosis owing to intolerance to treatment‐related toxicity.^[^ ^8^ ^]^

Similar to cancer cells in solid tumors, leukemic cells reprogram their metabolic pathways to support their proliferation and survival. Accumulating evidence demonstrates that AML and ALL cells in experimental animal models and patient tissue samples rely on glycolysis to sustain their relentless proliferative abilities.^[^ ^9^ ^]^ Consequently, targeting the glycolytic pathway in leukemic cells provides an attractive approach for treating acute leukemias.^[^ ^9^, ^10^ ^]^

Thermogenic adipose tissues (TATs), including brown adipose tissue (BAT) and browning white adipose tissue (bWAT), participate in energy dissipation by generating heat through non‐shivering thermogenesis (NST).^[^ ^11^ ^]^ In rodents, cold exposure and β3‐adrenoreceptor agonists augment NST by activation of TATs. Similarly, sympathetic activation of β3‐adrenoreceptor in adult humans by cold exposure or drugs also instigates NST in TATs.^[^ ^12^ ^]^ Our laboratory recently demonstrated that in experimental mice, TAT activation (TATA) markedly suppresses the growth of various solid tumors by attenuating glycolytic metabolism in cancer cells. TATA therapy provides a new therapeutic paradigm for the effective treatment of various cancers by altering global metabolism in cancer hosts.^[^ ^13^ ^]^ However, the impact of TATA therapy in both monotherapy and combination therapy settings on leukemias has not been investigated.

In this study, we examined the therapeutic effects of TATA in AML and ALL animal models. Our data show that TATA monotherapy markedly inhibited AML and ALL development and progression by impairing glycolysis in leukemic cells. Notably, combinations of TATA therapy with chemotherapy produced improved anti‐leukemic effects with lower toxicity. We believe that our findings pave a therapeutic concept for the effective treatment of AML, ALL, and potentially other types of hematological malignancies.

## 2 Results

### 2.1 Anti‐Leukemia Effects and Survival Improvement by Cold Exposure

**Figure 1:**
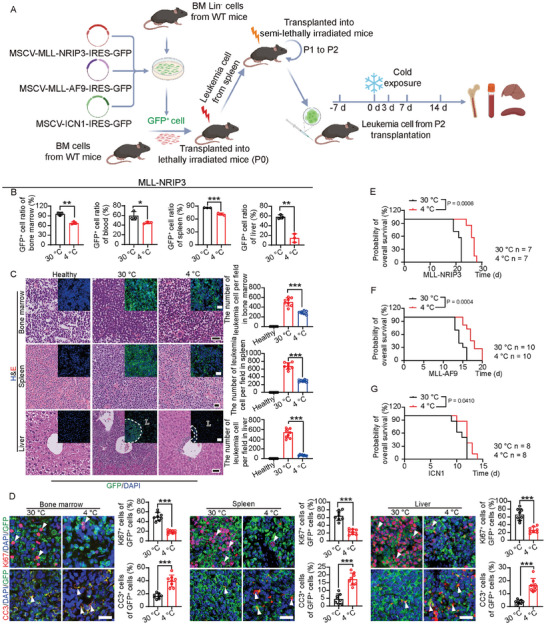
Cold exposure suppresses leukemia progression and prolongs survival in leukemia‐bearing mice. A) Schematic diagram of the establishment of leukemia models and cold exposure. After transplantation for 7 days, leukemia‐bearing mice were exposed to 30 or 4 °C for up to 14 days. B) Flow cytometry analysis of GFP^+^ cells in BM, PB, spleen, and liver in MLL‐NRIP3 leukemia‐bearing mice under 30 or 4  °C conditions (n  =  3 mice per group). C) Histological analysis and immunofluorescence analysis of BM, spleen, and liver in MLL‐NRIP3 leukemia‐bearing mice under 30 or 4 °C conditions (n  =  8 random fields per group). Healthy wt mice served as control. Quantification of infiltrated leukemia cells. Scale bar in upper and middle panels, 20 µm. Scale bar in the lower panel, 50 µm. Scale bar in fluorescence micrographs, 20 µm. L, liver. The dashed line indicates GFP^+^ leukemia cells. D) Immunofluorescence analysis of Ki67^+^ proliferating cells and cleaved caspase3^+^ apoptotic cells in BM, spleen, and liver in MLL‐NRIP3 leukemia‐bearing mice under 30  or 4  °C conditions (n  =  8 random fields per group). Scale bar, 20 µm. Arrowhead indicates positive signals. E–G) Overall survival of MLL‐NRIP3 leukemia‐bearing mice, MLL‐AF9 leukemia‐bearing mice, and ICN1 leukemia‐bearing mice under 30  or 4 °C conditions (n  =  7, 10, and 8 mice per group, respectively). ^*^ *p* < 0.05; ^**^ *p* < 0.01; ^***^ *p* < 0.001. NS = not significant. Data presented as mean ± s.d.

To study the effect of cold exposure on leukemic cell growth and survival, we employed three clinically relevant leukemia models in mice and examined the effects of cold acclimatization on leukemia cell growth, progression, and survival of leukemia‐bearing mice. The first leukemia model was a mouse genetic AML model. The translocation‐induced fusion protein, mixed lineage leukemia (MLL; also known as lysine methyltransferase 2A)‐nuclear receptor‐interacting protein 3 (NRIP3) is responsible for a subset of human AML and is capable of transforming progenitor cells into leukemia stem cells in mice.^[^ ^14^ ^]^ Murine hematopoietic stem and progenitor cells were transformed into AML cells by MLL‐NRIP3 using a murine stem cell virus (MSCV)‐based method. Similarly, ectopic expression of an MLL‐ALL1‐fused gene from chromosome 9 (AF9; also known as MLLT3 super elongation complex subunit) fusion protein was used as the second murine AML model.^[^ ^15^ ^]^ Additionally, the intracellular Notch1 domain (ICN1)‐driven ALL model was also deployed in our study.^[^ ^16^ ^]^ (**Figure** **1**A). All leukemia models were developed in lethally irradiated recipient mice by co‐transplantation of transduced BM Lin^−^ cells and BM nucleated cells. Primary GFP^+^ leukemic cells can be harvested, followed by transplantation into sublethally irradiated recipients for expansion, or into wt mice to establish leukemia models (Figure 1A).

After 1‐week transplantation, AML leukemia‐bearing immunocompetent C57BL/6 mice were randomly exposed to the thermoneutral temperature of 30 °C, or cold of 4 °C. Exposure of AML mice to cold for 14 days markedly inhibited the growth of GFP^+^ leukemia cells in BM, peripheral blood (PB), spleen, and liver (Figure S1A, Supporting Information). Along with the inhibition of leukemia cell proliferation, splenomegaly and hepatomegaly were significantly alleviated by reducing organ weights (Figure S1B, Supporting Information). On the basis of these findings, we chose a 2‐week cold exposure for subsequent experiments. In the MLL‐NRIP3‐AML model, cold exposure produced a significant anti‐leukemia effect by mitigating leukemic cells in BM, PB, liver, and spleen (Figure 1B). H&E analysis and detection of GFP^+^ cells corroborated the anti‐leukemic effects of cold exposure (Figure 1C). The cold‐triggered anti‐AML effect was further validated by decreasing the Ki67^+^ proliferating AML cell populations in various organs (Figure 1D). Significant increases in apoptotic AML cells were also detected by cold exposure. These data show that exposure of AML mice to cold significantly inhibits leukemic cell growth in vivo.

It is known that 4 °C cold exposure augments TATA in experimental mice by activation of the β3‐adrenoceptor sympathetic system.^[^ ^17^ ^]^ To further study the role of β3‐adrenoceptor activation in leukemic suppression, a specific β3‐adrenoceptor agonist, CL‐316243,^[^ ^18^ ^]^ was used to treat MLL‐NRIP3‐AML mice. Similar to cold exposure, CL‐316243 markedly inhibited leukemia progression and cell proliferation in vivo (Figure S1C–E, Supporting Information). These findings demonstrate that activation of the β3‐adrenoceptor sympathetic system offers a possible mechanism that underlies the cold‐triggered anti‐AML effect.

In addition to the MLL‐NRIP3‐AML model, 4 °C cold exposure also produced robust anti‐AML and anti‐ALL effects in the MLL‐AF9‐AML and ICN1‐ALL models (Figure S2, Supporting Information), suggesting that cold exposure‐mediated anti‐leukemia effects are likely generalized to other leukemia types. Consistent with lower proliferative and higher apoptotic rates, the survivals of three leukemia‐bearing models were markedly prolonged under 4 °C (Figure 1E–G). Together, these results demonstrate that exposure to cold or treatment with a β3‐adrenoceptor agonist significantly inhibits leukemia progression and improves survival in various leukemia models.

### 2.2 Inhibition of Leukemia Onset by Cold Exposure

**Figure 2:**
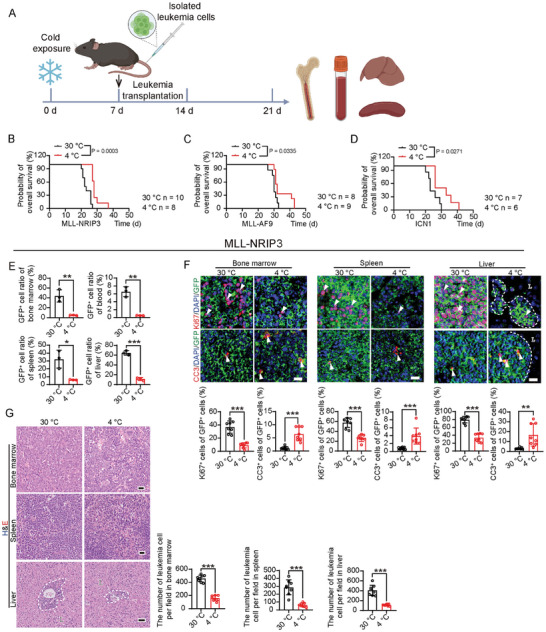
Pre‐treatment of cold prolongs survival in leukemia‐bearing mice. A) Schematic diagram of experimental design for testing the leukemia preventive effects of cold exposure. After 7 days of 30 or 4 °C exposure, mice were transplanted with leukemia and observed for up to 14 days. B–D) Overall survival of MLL‐NRIP3 leukemia‐bearing mice, MLL‐AF9 leukemia‐bearing mice, and ICN1 leukemia‐bearing mice under 30  or 4 °C conditions (n  =  6–10 mice per group). E) Flow cytometry analysis of GFP^+^ cells in BM, PB, spleen, and liver in MLL‐NRIP3 leukemia‐bearing mice under 30  or 4 °C conditions (n  =  3 mice per group). F) Histological analysis of BM, spleen, and liver in MLL‐NRIP3 leukemia‐bearing mice under 30 or 4 °C conditions (n  =  8 random fields per group). Quantification of infiltrated leukemia cells. Scale bar in upper and middle panels, 20 µm. Scale bar in the lower panel, 50 µm. G) Immunofluorescence analysis of Ki67^+^ proliferating cells and cleaved caspase3^+^ apoptotic cells in BM, spleen, and liver in MLL‐NRIP3 leukemia‐bearing mice under 30 or 4 °C conditions (n  =  8 random fields per group). Scale bar, 20 µm. Arrowhead indicates positive signals. L, liver. The dashed line indicates GFP^+^ leukemia cells. ^*^ *p* < 0.05; ^**^ *p* < 0.01; ^***^ *p* < 0.001. NS = not significant. Data presented as mean ± s.d.

We next investigated the effect of cold exposure on the onset of leukemia by pre‐exposure of healthy wild‐type animals to 4 °C cold. In this experimental setting, healthy wt mice were pre‐exposed to cold for 1 week prior to leukemic cell implantation (**Figure** **2**A). At the end of day 7, a timepoint known to fully activate TAT,^[^ ^19^ ^]^ AML and ALL leukemic cells were implanted into the 4 °C cold and 30 °C thermoneutral temperature‐pre‐exposed animals and assessed leukemia development at the end of day 21 (Figure 2A).

Interestingly, the overall survival of MLL‐NRIP3‐AML‐, MLL‐AF9‐AML‐, and ICN1‐ALL‐bearing mice were all significantly improved in cold‐pre‐exposed animals (Figure 2B–D). Given the aggressive nature of AML and ALL and the short lifetime between 20 and 30 days in these animal models, an extension of a lifetime of a few days was significant and meaningful. Consistent with survival improvement, leukemic cell proliferation and infiltration in BM, PB, spleen, and liver were markedly reduced (Figure 2E–G). These results provide convincing evidence that cold exposure markedly delays the onset of AML and ALL in mouse models.

### 2.3 BAT Removal and Genetic Deletion of *Ucp1* Obliterates Cold‐Triggered Anti‐Leukemic Effects

**Figure 3:**
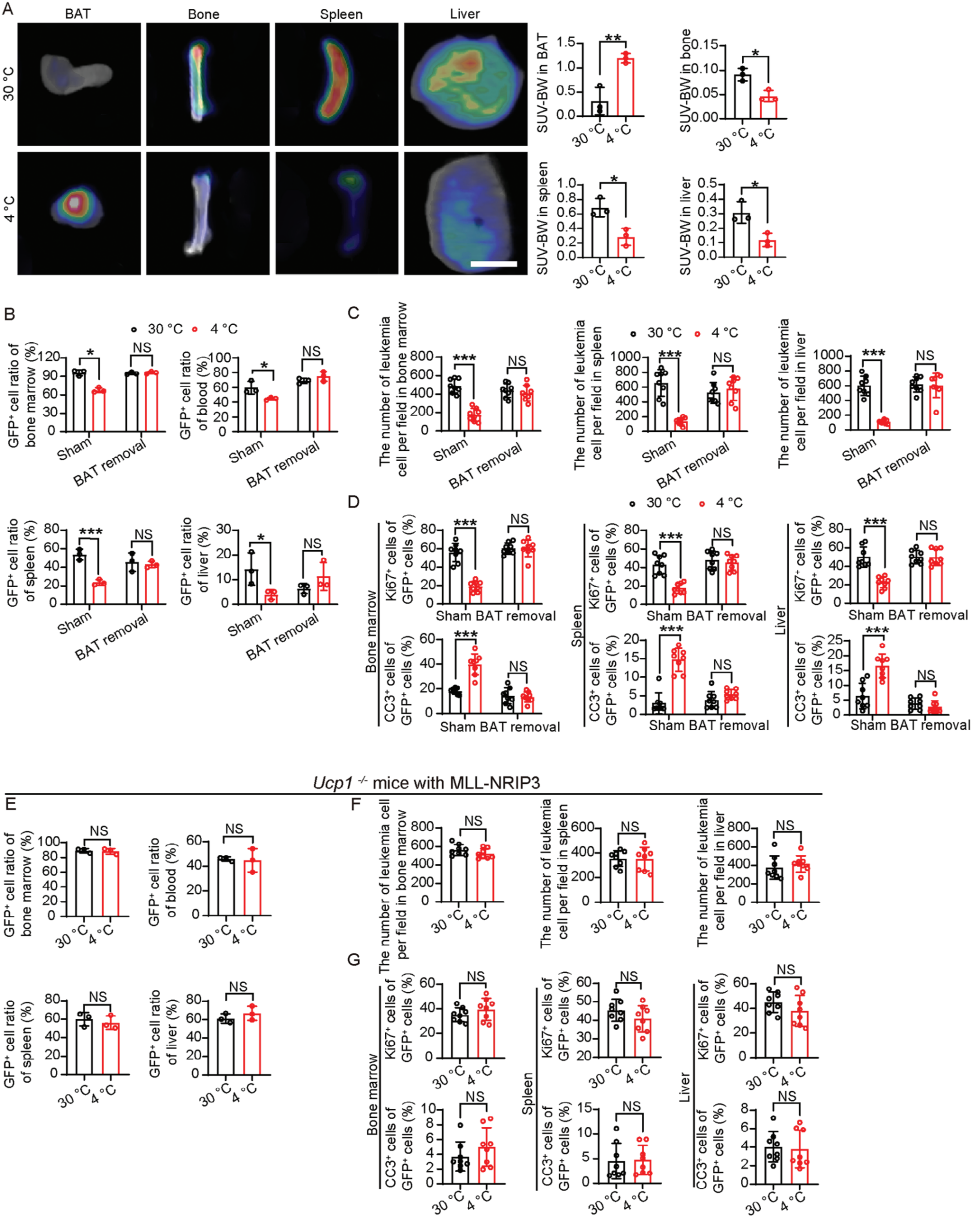
BAT removal or *Ucp1* deletion abolishes anti‐leukemia effect in vivo. A) Representative PET–CT images of BAT, femur bone, spleen, and liver. Scale bar 1 cm. Quantification of standardized uptake values (SUV) normalized to body weight (SUV‐BW). B) Flow cytometry analysis of GFP^+^ cells in BM, PB, spleen, and liver in sham‐operated or BAT‐removed MLL‐NRIP3 leukemia‐bearing mice under 30 or 4  °C conditions (n  =  3 mice per group). C) Quantification of infiltrated leukemia cells in BM, spleen, and liver in sham‐operated or BAT‐removed MLL‐NRIP3 leukemia‐bearing mice under 30 or 4  °C conditions (n  =  8 random fields per group). D) Quantification of Ki67^+^ proliferating cells and cleaved caspase3^+^ apoptotic cells in BM, spleen, and liver in sham‐operated or BAT‐removed MLL‐NRIP3 leukemia‐bearing mice under 30 or 4  °C conditions (n  =  8 random fields per group). E) Flow cytometry analysis of GFP^+^ cells in BM, PB, spleen, and liver in MLL‐NRIP3 leukemia‐bearing *Ucp1^−/−^* mice under 30 or 4  °C conditions (n  =  3 mice per group). F) Quantification of infiltrated leukemia cells in BM, spleen, and liver in MLL‐NRIP3 leukemia‐bearing *Ucp1^−/−^* mice under 30 or 4  °C conditions (n  =  8 random fields per group). G) Quantification of Ki67^+^ proliferating cells and cleaved caspase3^+^ apoptotic cells in BM, spleen, and liver in MLL‐NRIP3 leukemia‐bearing *Ucp1^−/−^* mice under 30 or 4  °C conditions (n  =  8 random fields per group). ^*^ *p* < 0.05; ^**^ *p* < 0.01; ^***^ *p* < 0.001. NS = not significant. Data presented as mean ± s.d.

To gain mechanistic insights into cold exposure‐induced anti‐leukemic effects, we focused on cold‐triggered TATA, which deploys increased glucose uptake. Similar to most solid tumors, leukemic cell growth is also dependent on glycolytic metabolism. For this reason, we monitored glucose distribution between BAT and leukemia‐evolved organs using the ^18^F‐fluorodeoxyglucose (^18^F‐FDG) PET‐CT imaging analysis. Expectedly, 4 °C cold exposure markedly activated BAT (Figure S3A, B, Supporting Information), and augmented ^18^F‐FDG uptake in BAT (**Figure** **3**A). Conversely, ^18^F‐FDG uptake was significantly reduced in bones, spleen, and liver under cold exposure (Figure 3A). To avoid the effect of uneven leukemic cells between the 30 °C‐ and 4 °C‐exposed groups, similar sizes of spleens and livers between the two groups were justified.

Since BAT constitutes a significant proportion of TAT, contributes to substantial glucose uptake, and becomes markedly activated under cold exposure, we therefore surgically removed BAT in leukemic mice. Notably, BAT removal neutralized the cold‐induced anti‐leukemic effects in the MLL‐NRIP3‐AML‐bearing mice (Figure 3B–D; Figure S3C,D, Supporting Information). UCP1 is known as one of the key thermogenic proteins in mitochondria. Knowing the crucial role of BAT in mediating the anti‐leukemia effect of cold exposure, we next deleted the *Ucp1* gene in mice. Similar to BAT removal, genetic deletion of *Ucp1* abolished the anti‐leukemic effect by cold exposure (Figure 3E–G; Figure S3E,F, Supporting Information).

Independent evidence of TATA‐mediated anti‐leukemic effects was obtained using the MLL‐AF9‐AML in combination with BAT removal or *Ucp1* knockout models (Figure S4A–F, Supporting Information). More importantly, BAT removal neutralized the survival improvement by cold exposure (Figure S4G, Supporting Information). These findings provide compelling evidence to support the cold exposure‐augmented TATA in mediating anti‐leukemic effects.

### 2.4 Deterioration of Glycolysis in Leukemia Cells by Cold Exposure

**Figure 4:**
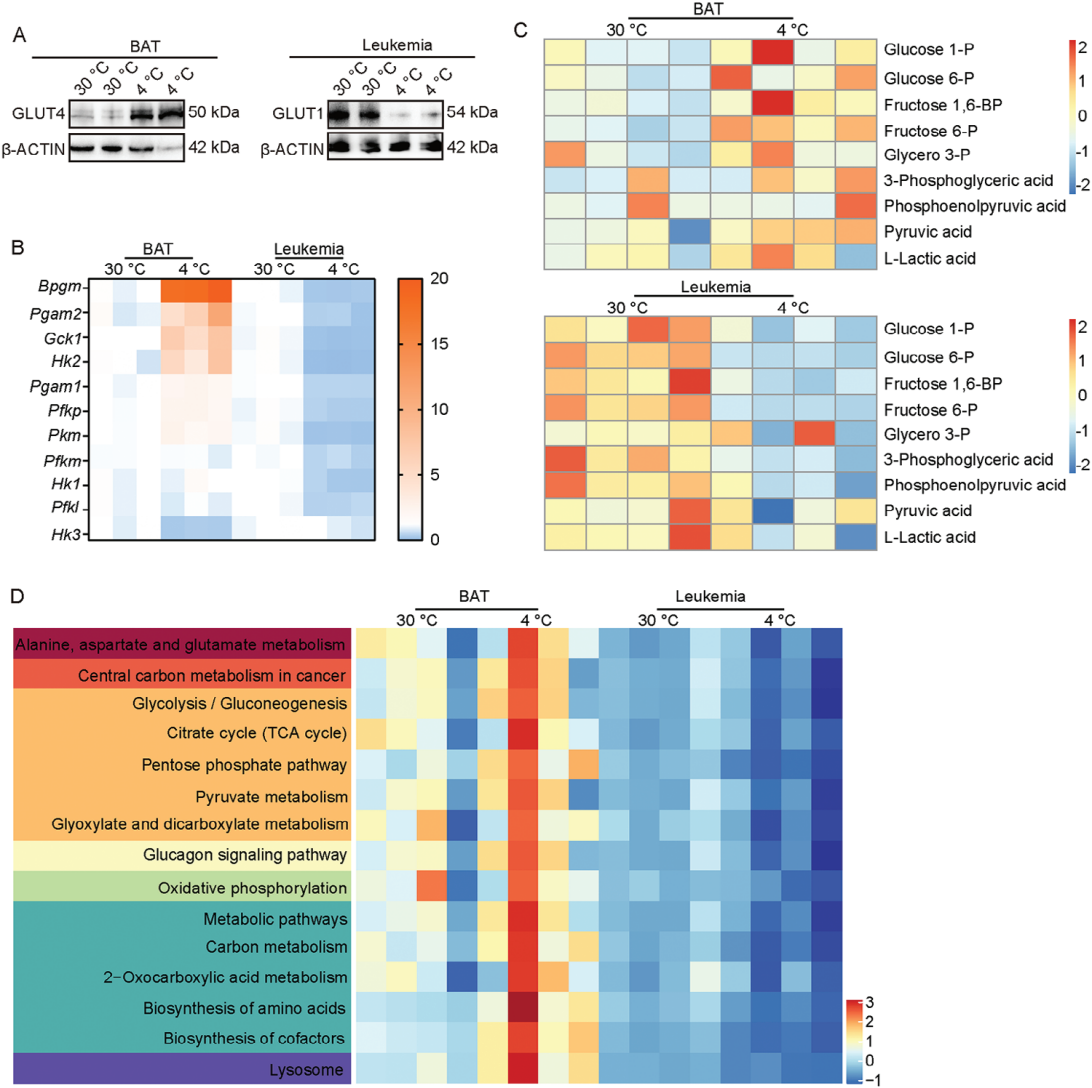
Glucose metabolism in leukemia sites and TAT. A) After transplantation for 7 days, leukemia‐bearing mice were exposed to 30 °C or 4 °C for up to 14 days. Western blot of GLUT4 in BAT and GLUT1 in leukemia infiltrated spleen tissues in MLL‐NRIP3 leukemia‐bearing mice under 30 or 4  °C conditions. B) Quantitative PCR array of glucose metabolism‐related genes in BAT and leukemia infiltrated spleen tissues in MLL‐NRIP3 leukemia‐bearing mice under 30 or 4  °C conditions (n  =  3 mice per group). C) Metabolomic heat‐map analysis of glycolysis‐related metabolites in BAT and leukemia infiltrated spleen tissues in MLL‐NRIP3 leukemia‐bearing mice under 30 or 4  °C conditions (n  =  4 mice per group). D) Pathway analysis of glycolysis‐related metabolites in BAT and leukemia infiltrated spleen tissues in MLL‐NRIP3 leukemia‐bearing mice under 30 or 4  °C conditions (n  =  4 mice per group). ^*^ *p* < 0.05; ^**^ *p* < 0.01; ^***^ *p* < 0.001. NS = not significant. Data presented as mean ± s.d.

The cold exposure‐mitigated glucose uptake in leukemic cells suggested that impaired glycolysis might be entailed in leukemia suppression. We analyzed the glycolytic pathways of metabolism in leukemia‐infiltrated spleen tissues and BAT under cold and thermoneutral exposure conditions. After transplantation for 7 days, leukemia‐bearing mice were exposed to 30 or 4 °C for up to 14 days. Cold exposure of MLL‐NRIP3‐AML‐bearing mice markedly increased glucose transporters GLUT4 in BAT (**Figure** **4**A). By contrast, GLUT1 levels were markedly reduced in leukemic cells (Figure 4A). Consistently, qPCR‐array analysis of glycolysis‐related enzymic genes showed a marked increase in glycolysis‐related gene expression in BAT (Figure 4B). In contrast, the expression of these genes was markedly downregulated in leukemic cells (Figure 4B). Reconciling with changes in glycolytic enzymes, glycolytic metabolites in BAT and leukemic cells were accordingly altered (Figure 4C). Further pathway enrichment analysis of metabolomics showed that glutamate metabolism, carbon metabolism, glycolysis, oxidative phosphorylation, and biosynthesis were boosted in BAT, but markedly mitigated in leukemic cells after cold exposure (Figure 4D). These results support that cold exposure alters metabolic pathways in leukemic cells by suppressing glycolytic metabolism and global metabolic changes in TAT.

### 2.5 Improvement of Anti‐Leukemia Effects of Chemotherapy

**Figure 5:**
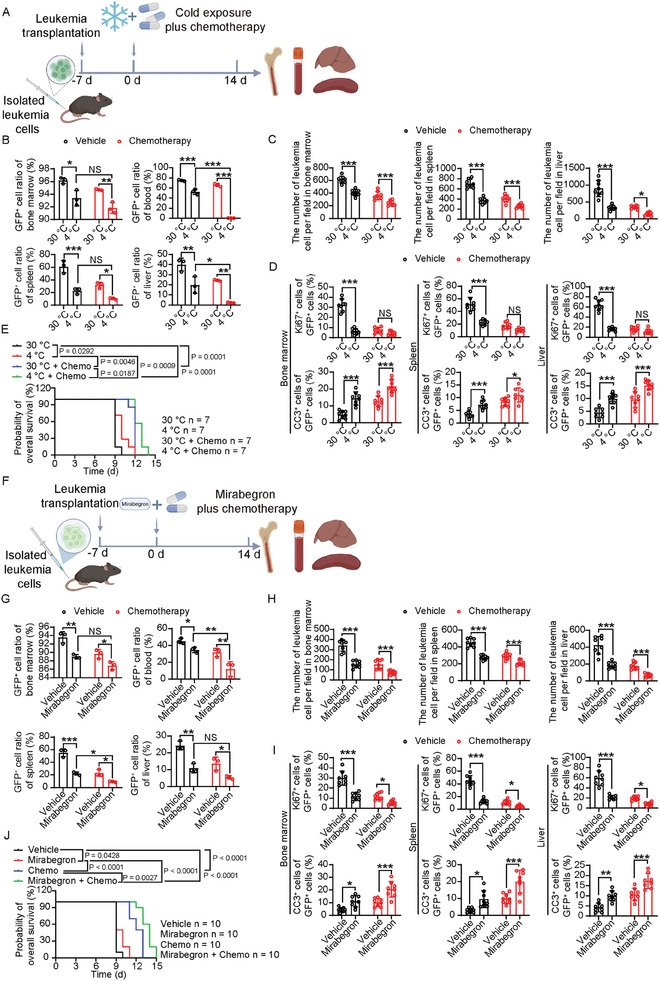
Combination of TATA therapy and chemotherapeutics synergistically suppresses AML progression. A) Schematic diagram of experimental design for testing anti‐leukemia effects of combination therapy of chemo drugs and cold exposure. After transplantation for 7 days, leukemia‐bearing mice were treated with combination therapy for up to 14 days. B) Flow cytometry analysis of GFP^+^ cells in BM, PB, spleen, and liver in vehicle‐ or chemo‐treated MLL‐NRIP3 leukemia‐bearing mice under 30 or 4  °C conditions (n  =  3 mice per group). C) Quantification of infiltrated leukemia cells in BM, spleen, and liver in vehicle‐ or chemo‐treated MLL‐NRIP3 leukemia‐bearing mice under 30 or 4  °C conditions (n  =  8 random fields per group). D) Quantification of Ki67^+^ proliferating cells and cleaved caspase3^+^ apoptotic cells in BM, spleen, and liver in vehicle‐ or chemo‐treated MLL‐NRIP3 leukemia‐bearing mice under 30 or 4  °C conditions (n  =  8 random fields per group). E) Overall survival of vehicle‐ or chemo‐treated MLL‐NRIP3 leukemia‐bearing mice under 30 or 4  °C conditions (n  =  7 mice per group). F) Schematic diagram of experimental design for testing anti‐leukemia effects of combination therapy of chemo drugs and mirabegron. After transplantation for 7 days, leukemia‐bearing mice were treated with combination therapy for up to 14 days. G) Flow cytometry analysis of GFP^+^ cells in BM, PB, spleen, and liver in vehicle‐ or chemo‐treated MLL‐NRIP3 leukemia‐bearing mice under vehicle or mirabegron treatment (n  =  3 mice per group). H) Quantification of infiltrated leukemia cells in BM, spleen, and liver in vehicle‐ or chemo‐treated MLL‐NRIP3 leukemia‐bearing mice under vehicle or mirabegron treatment (n  =  8 random fields per group). I) Quantification of Ki67^+^ proliferating cells and cleaved caspase3^+^ apoptotic cells in BM, spleen, and liver in vehicle‐ or chemo‐treated MLL‐NRIP3 leukemia‐bearing mice under vehicle or mirabegron treatment (n  =  8 random fields per group). J) Overall survival of vehicle‐ or chemo‐treated MLL‐NRIP3 leukemia‐bearing mice under vehicle or mirabegron treatment (n  =  10 mice per group). ^*^ *p* < 0.05; ^**^ *p* < 0.01; ^***^ *p* < 0.001. NS = not significant. Data presented as mean ± s.d.

To test the possible anti‐leukemic effects of TATA therapy in combination with chemotherapy, which is a standard treatment for AML, we applied a standard chemotherapeutic regimen containing the clinically used drugs, cytarabine and doxorubicin, along with TATA therapy (**Figure** **5**A). In the MLL‐NRIP3‐AML model, a combination of cold exposure with the standard chemotherapy produced a significant enhancement of the anti‐leukemic effect (Figure 5B–D; Figure S5A,B, Supporting Information). It should be emphasized that chemotherapy alone achieved a robust anti‐proliferative effect in leukemic cells, which could not be further enhanced by cold exposure (Figure 5D). However, the number of apoptotic cells was increased in cold exposure combined with chemotherapy relative to those of the 30 °C with chemotherapy (Figure 5D). These data demonstrate that cold exposure significantly enhanced the anti‐leukemic effect of chemotherapy. Concordantly, the combination of cold exposure plus chemotherapy significantly improved the survival of MLL‐NRIP3‐AML‐bearing mice (Figure 5E). The enhanced anti‐leukemia effect of cold exposure plus chemotherapy was further validated using the MLL‐AF9‐AML model (Figure S5C–F, Supporting Information). These data demonstrate that a combination of cold exposure with chemotherapy produces improved anti‐leukemic effects and prolongs the survival of AML‐bearing mice.

To study the impact of the combination of the TAT‐activating β3‐adrenoceptor agonists with chemotherapy, we used mirabegron, a clinically approved drug for treating overactive bladder. Similar to CL‐316243, mirabegron alone exhibited a potent anti‐leukemic effect (Figure 5F–I; Figure S6A,B, Supporting Information), which was equivalent to that of cold exposure (Figure 1). In a combination setting, mirabegron with standard chemotherapy improved therapeutic outcomes, including reducing leukemic cells, organ infiltration, and survival improvement (Figure 5F–J; Figure S6A,B, Supporting Information). These data indicate that a combination of the TAT‐activating β3‐adrenoceptor agonists with chemotherapy provides a new therapeutic paradigm for the effective treatment of leukemias.

### 2.6 Mitigating Chemotoxicity by a Combination of TATA Therapy with a Low‐Dose Chemotherapy

**Figure 6:**
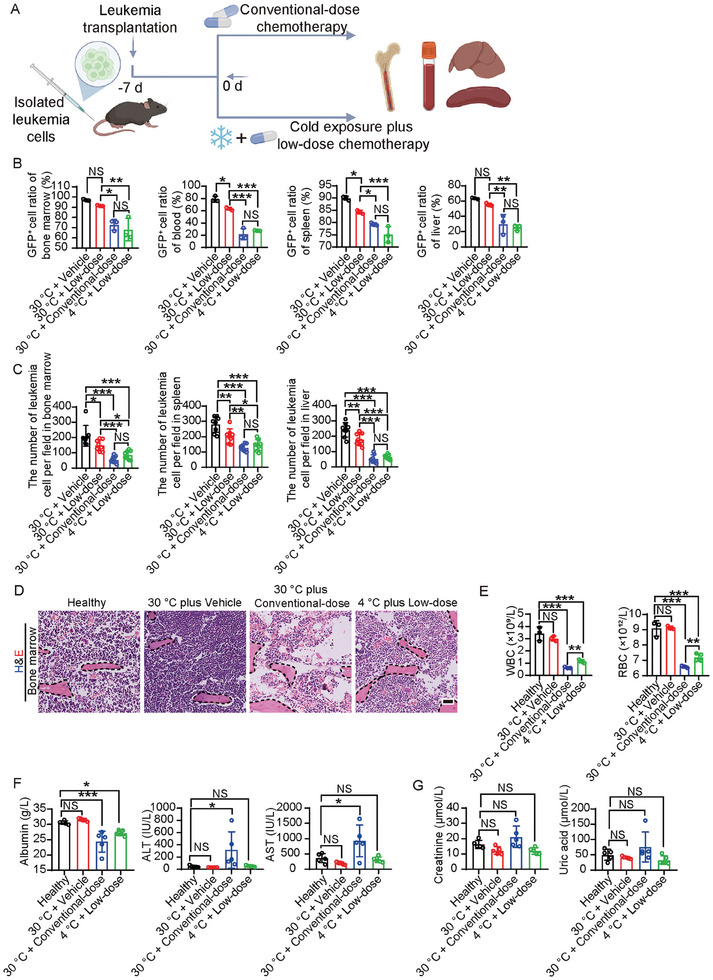
Toxicity of conventional chemotherapy and combination therapy. A) Schematic diagram of experimental design for testing side effects of conventional chemotherapy and the combination therapy of low‐dose chemo drugs and cold exposure. After transplantation for 7 days, leukemia‐bearing mice were treated with combination therapy or chemotherapy for up to 14 days. B) Flow cytometry analysis of GFP^+^ cells in BM, PB, spleen, and liver in vehicle‐, low‐dose chemotherapy‐, conventional chemotherapy‐ or combination therapy‐treated MLL‐NRIP3 leukemia‐bearing mice (n  =  3 mice per group). C) Quantification of infiltrated leukemia cells in BM, spleen, and liver in vehicle‐, conventional chemotherapy‐ or combination therapy‐treated MLL‐NRIP3 leukemia‐bearing mice (n  =  8 random fields per group). D) Histology analysis of BM in vehicle‐, conventional chemotherapy‐ or combination therapy‐treated MLL‐NRIP3 leukemia‐bearing mice. Healthy BM served as control. Scale bar, 50 µm. Dashed line marked trabecular bone. E) RBC and WBC levels in vehicle‐, conventional chemotherapy‐ or combination therapy‐treated MLL‐NRIP3 leukemia‐bearing mice (n  =  3–4 mice per group). F) Serum albumin, serum alanine aminotransferase, and serum aspartate transaminase levels in conventional chemotherapy‐ or combination therapy‐treated MLL‐NRIP3 leukemia‐bearing mice. Serum of healthy mice served as control (n  =  5 mice per group). G) Serum creatinine and uric acid levels in conventional chemotherapy‐ or combination therapy‐treated MLL‐NRIP3 leukemia‐bearing mice. Serum of healthy mice served as control (n  =  5 mice per group). ^*^ *p* < 0.05; ^**^ *p* < 0.01; ^***^ *p* < 0.001. NS = not significant. Data presented as mean ± s.d.

The enhanced anti‐leukemic effects of TATA therapy with standard chemotherapy encouraged us to test the possibility of reducing the dose of chemotherapy to achieve our goal of mitigating chemotoxicity without jeopardizing its anti‐leukemia effect. For this purpose, we chose a half‐dose of the standard chemotherapy in combination with cold exposure (**Figure** **6**A). Under the thermoneutral condition, the half‐dose of chemotherapy produced an incremental effect against leukemia (Figure 6B,C). However, the combination of cold exposure with the half‐dose of chemotherapy produced a marked increase in the anti‐leukemia effect, which was indistinguishable from that of the full dose of chemotherapy alone (Figure 6B,C). The combination of cold exposure with the low dose of chemotherapy produced markedly reduced chemotoxicity, including BM toxicity, white blood cell (WBC) and red blood cell (RBC) counts, liver toxicity, renal toxicity, body weight, and food intake (Figure 6D–G; Figure S7A,B, Supporting Information). These results show that cold exposure reduces chemotoxicity for the treatment of leukemia.

### 2.7 Prolonged Remission of AML by Sustained Cold Exposure Plus Chemotherapy

**Figure 7:**
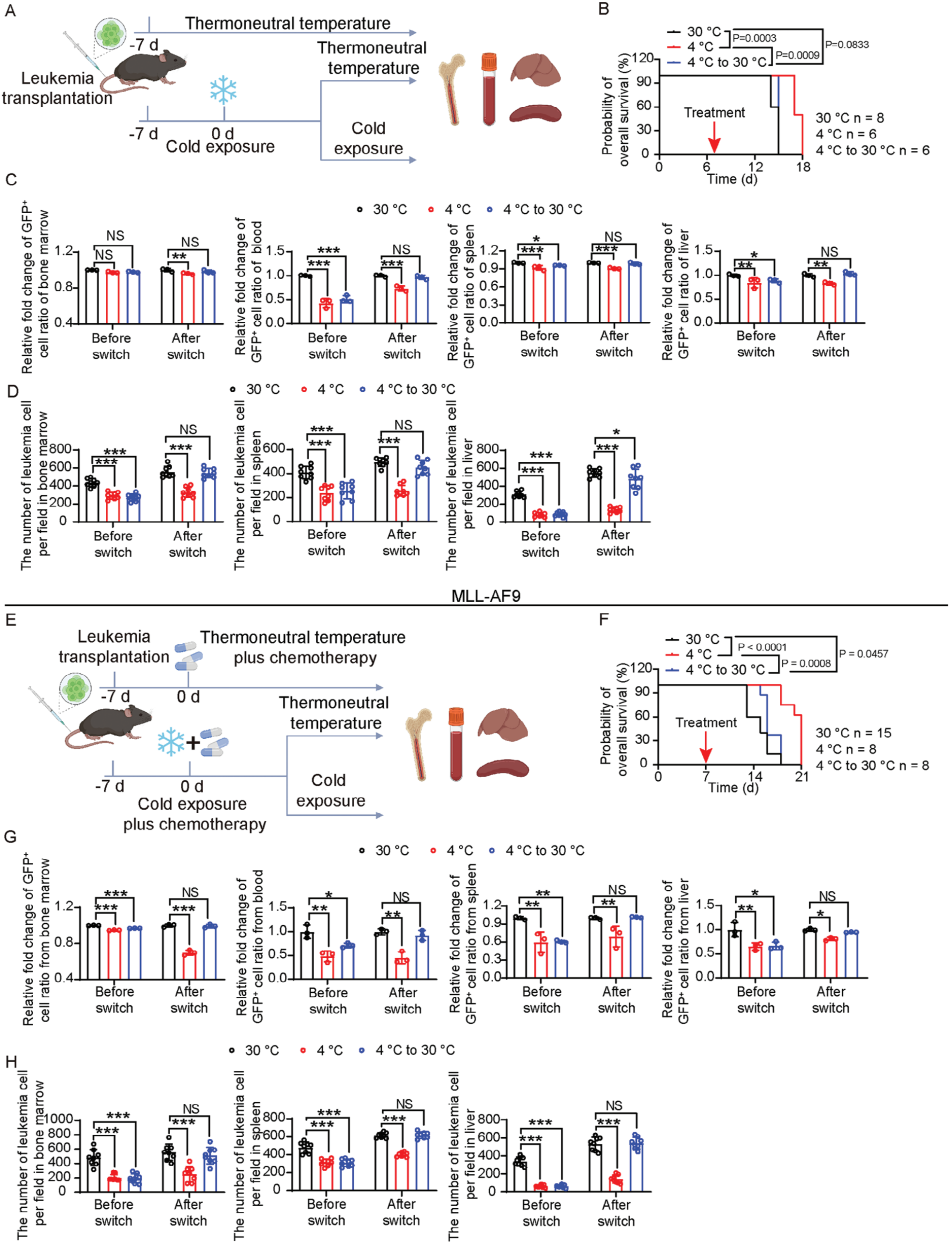
Long‐term remission of AML by continuous cold exposure. A) Schematic diagram of experimental design for testing anti‐leukemia effects of continuous cold exposure. After transplantation for 7 days, leukemia‐bearing mice were treated with TATA therapy for 7 days. Mice were then switched to thermoneutral temperature or continued for TATA therapy. B) Overall survival of 30  °C‐, 4  °C‐, or 4  °C‐ to −30  °C‐ exposed MLL‐NRIP3 leukemia‐bearing mice (n  =  6–8 mice per group). C) Flow cytometry analysis of GFP^+^ cells in BM, PB, spleen, and liver in 30  °C‐, 4  °C‐, or 4  °C‐ to −30  °C‐ exposed MLL‐NRIP3 leukemia‐bearing mice (n  =  3 mice per group). D) Quantification of infiltrated leukemia cells in BM, spleen, and liver in 30  °C‐, 4  °C‐, or 4  °C‐ to −30  °C‐ exposed MLL‐NRIP3 leukemia‐bearing mice (n  =  8 random fields per group). E) Schematic diagram of experimental design for testing anti‐leukemia effects of continuous cold exposure in combination therapy. After transplantation for 7 days, leukemia‐bearing mice were treated with combination therapy for 7 days. Mice were then switched to chemotherapy alone or continued for combination therapy. F) Overall survival of 30  °C‐, 4  °C‐, or 4  °C‐ to −30  °C‐ exposed, low‐dose chemotherapy‐treated MLL‐AF9 leukemia‐bearing mice (n  =  8‐15 mice per group). G) Flow cytometry analysis of GFP^+^ cells in BM, PB, spleen, and liver in 30  °C‐, 4  °C‐, or 4  °C‐ to −30  °C‐ exposed, low‐dose chemotherapy‐treated MLL‐AF9 leukemia‐bearing mice (n  =  3 mice per group). H) Quantification of infiltrated leukemia cells in BM, spleen, and liver in 30  °C‐, 4  °C‐, or 4  °C‐ to −30  °C‐ exposed, low‐dose chemotherapy‐treated MLL‐AF9 leukemia‐bearing mice (n  =  8 random fields per group). ^*^ *p* < 0.05; ^**^ *p* < 0.01; ^***^ *p* < 0.001. NS = not significant. Data presented as mean ± s.d.

To study the impact of cold exposure plus chemotherapy on leukemia remission, we performed On/Off drug experiments in TATA monotherapy and combination therapy settings (**Figure** **7**A). After transplantation for 7 days, leukemia‐bearing mice were treated with TATA therapy for 7 days. Mice were then switched to thermoneutral temperature or continued for TATA therapy. First, we noticed that sustained cold exposure was required to achieve survival improvement in AML‐bearing mice, and discontinuation ablated this effect (Figure 7B). Consistent with survival improvement, leukemic cell proliferation and invasion also required sustained exposure to cold (Figure 7C,D; Figure S8A, Supporting Information). In the combination therapy setting, sustained cold exposure was also required to maintain the anti‐leukemic effect (Figure 7E–H; Figure S8B, Supporting Information). These data show that sustained cold exposure is required for the maintenance of anti‐leukemic effects.

### 2.8 TATA Therapeutic Effects in Clinically Relevant Human Leukemia Models

**Figure 8:**
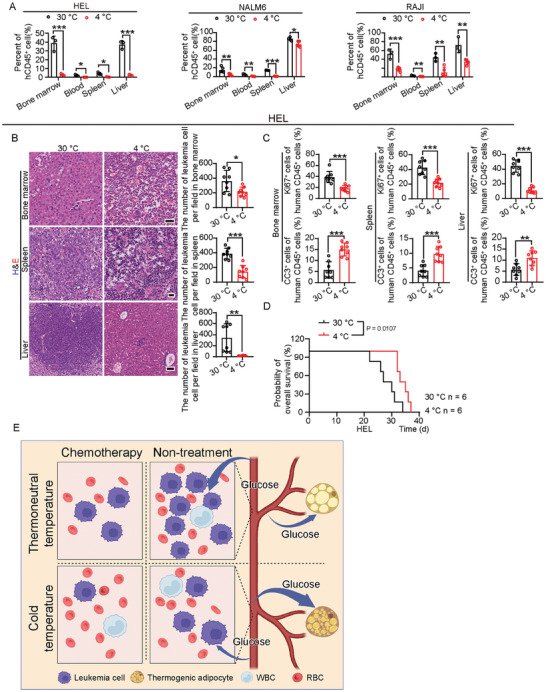
Cold exposure suppresses leukemia progression and prolongs survival in human leukemia xenografts. A) Flow cytometry analysis of human CD45^+^ cells in BM, PB, spleen, and liver in HEL‐bearing mice, NALM6‐bearing mice, and RAJI‐bearing mice under 30 or 4 °C conditions (n  =  3 mice per group). B) Histology and quantification of infiltrated leukemia cells in BM, spleen, and liver in HEL‐bearing mice under 30 or 4  °C conditions (n  =  8 random fields per group). Scale bar in upper and middle panels, 20 µm. Scale bar in lower panel, 50 µm. C) Immunofluorescence analysis of Ki67^+^ proliferating cells and cleaved caspase3^+^ apoptotic cells in BM, spleen, and liver in HEL‐bearing mice under 30 or 4  °C conditions (n  =  8 random fields per group). D) Overall survival of HEL‐bearing mice under 30 or 4  °C conditions (n  =  6 mice per group). E) Schematic diagram of mechanisms underlying cold exposure‐induced leukemia suppression. Cold exposure induces TATA with significant glucose uptake and thermogenesis. Consequently, leukemia progression under cold exposure is inhibited via global glucose redistribution and glucose metabolism inhibition. Combining cold exposure with low‐dose chemotherapy has a robust anti‐leukemia effect and low toxicity such as anemia and leukopenia, which is beneficial to patients with leukemia. ^*^ *p* < 0.05; ^**^ *p* < 0.01; ^***^ *p* < 0.001. NS = not significant. Data presented as mean ± s.d.

To investigate the clinical relevance of our findings in leukemia mouse models, we performed TATA therapy in human leukemia xenograft models. Immunodeficient B‐NDG mice were irradiated and transplanted with human HEL‐AML cells, human NALM6‐ALL cells, and human RAJI‐lymphoma cells. Human leukemia or lymphoma‐bearing mice were exposed to 4 °C cold. Similar to mouse leukemic models, human CD45^+^ leukemia cells in BM, PB, spleen, and liver were significantly inhibited in all three human leukemia models (**Figure** **8**A). Histochemistry analysis revealed that cold exposure rescued organ architecture in BM, spleen, and liver in all three human leukemia models (Figure 8B; Figure S9A,B, Supporting Information). Detection of CD45^+^ cells confirmed that cold exposure inhibited the proliferation of leukemia cells and increased apoptosis in the human leukemia model (Figure 8C). Cold exposure significantly prolonged the overall survival of HEL‐AML‐bearing mice (Figure 8D). These results demonstrate the clinical relevance of our findings and thus can be implicated in the clinical practice of our therapy.

## 3 Discussion

Owing to their poor clinical response to current therapies and prognosis, the development of novel therapies for the effective treatment of acute leukemias is urgently needed. In this study, we provide compelling evidence to demonstrate that TATA therapy is effective for the treatment of acute leukemias in mouse models. Unlike other anti‐leukemic drugs,^[^ ^20^ ^]^ the underlying mechanism of TATA therapy is to alter the global thermogenic metabolism in cancer hosts rather than directly targeting leukemic cells. TATA by cold exposure or β3‐adrenoceptor agonists is well‐tolerated in experimental animals without causing adverse effects. Similarly, cold exposure and treatment with mirabegron, an FDA‐approved anti‐overactive bladder drug,^[^ ^18^, ^21^ ^]^ sufficiently activate TATs in adult humans. Thus, our findings in adult mouse models are clinically relevant.

In contrast to solid tumors, leukemia cells invade multiple tissues and organs, including BM, liver, spleen, and PB. In this regard, leukemias resemble a systemic and metastatic cancer disease that affects multiple tissues.^[^ ^22^ ^]^ Thus, localized therapeutic approaches that target cancer masses are less likely to be effective, and systemic therapeutic regimens are desirable. Similar to cancer cells in solid tumors, the proliferation of leukemic cells is also dependent on reprogrammed metabolic pathways, often activation of aerobic glycolysis as a commonly shared metabolic pathway in cancer cells for energy production.^[^ ^9^, ^10^ ^]^ Targeting reprogrammed glycolytic metabolism provides a new and exciting opportunity for treatment and prevention of various leukemias, including the most common, aggressive, and hard‐to‐treat AML and ALL. Recent studies have shown that inhibition of AML glycolysis by 2‐deoxy‐D‐glucose (2‐DG), a glycolysis inhibitor, produced a potent anti‐leukemia effect.^[^ ^9d^ ^]^ Similarly, a low‐dose 2‐DG therapy eliminated ALL leukemic cells and reversed drug resistance.^[^ ^23^ ^]^

Our recent study shows that TATA in mouse tumor models has potent anti‐cancer activity against a myriad of cancer types, including the most aggressive and hard‐to‐treat cancers such as pancreatic ductal adenocarcinoma and hepatocellular carcinoma.^[^ ^13^ ^]^ Although complex molecular mechanisms underlying TATA anti‐cancer therapy remain elusive, TATA‐triggered impairment of glycolysis in cancer cells has been observed. One of the possible mechanisms is that TATA increases glucose uptake in TAT adipocytes and thus competitively mitigates glucose availability in cancer cells. If so, the same mechanism would also retard glycolytic metabolism in leukemia cells, which is the key metabolic pathway for anabolic cell growth. Inhibition of tumor glycolysis drives SIRT3‐mediated metabolic paradigm shifts in other tumor types.^[^ ^24^ ^]^ Whether leukemias share this interesting mechanism requires further investigation. Indeed, we show that TATA therapy alone is markedly effective for the treatment and prevention of AML and ALL in mice (Figure 8E).

We show that TATA‐induced anti‐leukemia effect is dependent on the activation of BAT. Surgical removal of BAT largely obliterated the anti‐leukemia effects. Similarly, the genetic deletion of UCP1, the key component for mediating NST, also abolished the suppression of acute leukemias. On this basis, we speculate that the TATA‐induced anti‐leukemia effect is age‐dependent, as TATA declines with age.^[^ ^25^ ^]^ We should emphasize that other critical components for the futile creatine cycle, including creatine kinase B (CKB) and glycine amidinotransferase (GATM), the rate‐limiting enzyme of creatine biosynthesis, are also essential for adipose thermogenesis.^[^ ^26^ ^]^ Genetic deletion of CKB or GATM in adipocytes diminishes adipose thermogenic activity and predisposes to obesity and insulin resistance. The role of CKB or GATM in the mediation of TATA‐induced anti‐leukemia effects warrants future study.

One of the important discoveries of this study is that a combination of TATA therapy with chemotherapy could produce enhanced anti‐leukemic effects in both AML and ALL models. In particular, low‐dose chemotherapy combined with TATA therapy displays enhanced effects on leukemia suppression. TATA sensitizes chemotherapeutic effects against leukemias. Taking the severe adverse effects of high‐dose chemotherapy into account,^[^ ^27^ ^]^ the standard chemotherapy‐based therapeutic regimens for leukemias are not tolerated by all patients. In fact, a majority of elderly AML and ALL patients do not tolerate the standard chemotherapies.^[^ ^28^ ^]^ Because of the intolerable toxicities, current standard therapies provide only limited therapeutic benefits. Thus, a combination of TATA therapy with low‐dose chemotherapy without causing severe adverse effects would be desirable for the treatment of leukemias.

Although we provide experimental evidence of AML and ALL as two examples, our therapeutic concept is likely to be valid for treating other types of hematological malignancies, including chronic myeloid leukemia (CML), chronic lymphocytic leukemia (CLL), and lymphomas.^[^ ^29^ ^]^ These cancer cells are also dependent on reprogrammed metabolism for their anabolic cell growth. If so, our TATA therapy has broad implications for effective treatment of various leukemias.

## 4 Experimental Section

### Cell Culture

Human AML cell line HEL, human ALL cell line RAJI, and human ALL cell line NALM6 were obtained from the State Key Laboratory of Experimental Hematology's Blood Biobank, Tianjin, China, and were cultured in 10% FBS‐Roswell Park Memorial Institute (RPMI) 1640 medium (Cat. No. 10099141C, Thermo Fisher Scientific; Cat. No. C11875500BT, Thermo Fisher Scientific) supplemented with 100 U mL^−1^ penicillin, 100 µg mL^−1^ streptomycin (Cat. No. SV30010, HyClone). All cell lines used in the study were negative for mycoplasma confirmed by a MycAway Plus‐Color One‐Step Mycoplasma Detection Kit (Cat. No. 40612ES25, YEASEN, China).

### Animals

All animal experiments were approved by the Animal Experimental Ethical Committee of Fudan University, Shanghai, China (20221221‐008) or by the Institutional Animal Care and Use Committee at the Institute of Hematology, Chinese Academy of Medical Sciences, Tianjin, China (CIFMS2021004‐EC‐2). Male C57BL/6 mice and *Ucp1^−/−^* mice in the C57BL/6 background at the age between 6 and 10‐week‐old were purchased from GemPharmatech, China, and were maintained under 12‐h dark/12‐h light cycle with food and water provided ad libitum. Female NOD.CB17‐*Prkdc^scid^Il2rg^1^*/Bcgen (B‐NDG) immunodeficient mice at the age between 8 and 9‐week‐old were purchased (Cat. No. 110586, Biocytogen, China). All animals were randomly assigned to groups before experiments. The experimenter was not blinded to group allocation and results assessment. No statistical methods were used to predetermine the sample size. No samples, animals, or data were excluded.

### Leukemia Mouse Models

The MSCV‐MLL‐NRIP3‐GFP, MSCV‐MLL‐AF9‐GFP, or MSCV‐ICN‐IRES‐GFP construct was transfected into 293T cells together with packaging vectors pKAT and pCMV‐VSV‐G using Lipofectamine 2000 transfection reagent (Cat. No. 11668019, Thermo Fisher Scientific). Virus‐containing conditioned medium was collected after 72 h. BM cells from 6 to 8 week C57BL/6J mice were harvested. RBCs were removed using RBC Lysis Buffer (Cat. No. 00‐4333‐57, Thermo Fisher Scientific) and BM Lin^−^ cells were sequentially isolated using a mouse Direct Lineage Cell Depletion Kit (Cat. No. 130‐110‐470, Miltenyi Biotec). BM Lin^−^ cells (5 × 10^5^ per well) were seeded in a 24‐well plate pretreated with retronectin (Cat. No. T100A, Takara), and were cultured in IMDM (Cat. No. C12440500BT, Thermo Fisher Scientific) supplemented with 10% FBS, 50 ng mL^−1^ mouse recombinant SCF (Cat. No. 78064.1, STEMCELL Technologies), 100 ng mL^−1^ mouse recombinant Flt3/Flk‐2 ligand (Cat. No. 78011.1, STEMCELL Technologies), and 50 ng mL^−1^ mouse recombinant TPO (Cat. No. 78072.1, STEMCELL Technologies). Cells were cultured with the virus‐containing medium for 8 h and then changed to a fresh medium for 48 h.

Recipient 8‐week‐old C57BL/6 mice were irradiated with a lethal dose of X‐ray (123 cGy min^−1^, 4.8 Gy per time, twice with an interval of 4 h). After 4 h of irradiation, 5 × 10^5^ GFP^+^ cells were sorted out from transfected BM Lin^−^ cells, mixed with 2 × 10^5^ BM nucleated cells, and transplanted i.v. into each recipient mouse. For leukemia cell expansion, 5 × 10^5^ primary GFP^+^ leukemia cells were isolated by FACS from the spleen of end‐stage primary leukemia mice and transplanted into sublethally irradiated (4.8 Gy) recipient mice. After two generations, leukemia cells can be continuously expanded in sublethally irradiated recipient mice or 1 × 10^6^ primary GFP^+^ leukemia cells be transplanted i.v. into wt mice to establish leukemia models.

### Cold Exposure

Mice caged at room temperature were randomly divided into two groups for 30 °C exposure and 4 °C exposure, and were housed in open cages in the climate chamber (Cat. No. HWS‐350FT, Binglin electronics, China; or Cat. No. RGX‐180A, Chuanhong electronics, China). C57BL/6 mice were adapted at 16 °C for one night before exposure to a cold temperature of 4 °C. After 10–17 days of human leukemia cell transplantation, B‐NDG mice were adapted to a gradient temperature from 18 to 4 °C for 4 days before exposure to a cold temperature of 4 °C. For the thermoneutral group, mice were kept at 30 °C for the duration equivalent to cold exposure.

### Mouse Models

For BAT removal, mice were anesthetized by isoflurane (Cat. No. R510‐22, RWD Life Science, China) using a Compact Small Animal Anesthesia Device (Cat. No. R500IE, RWD Life Science, China). The BAT was surgically exposed by blunt dissection and was removed without compromising the blood supply to surrounding tissues. In the sham operation, adipose tissues were exposed but not excised. After wound closure, animals were kept at 30 °C until recovery from the operation. For human leukemia xenograft models, female B‐NDG mice were irradiated with 1.0 Gy X‐ray, and after 1 day, 5 × 10^5^ HEL cells, 5 × 10^4^ RAJI cells, or 5 × 10^4^ NALM6 cells were transplanted i.v. into each recipient mouse. In some of the experiments, a complete blood count was performed.^[^ ^30^ ^]^ Blood samples were collected into anticoagulation tubes and examined by an automated hematology analyzer (Cat. No. BC‐2600, Mindary, China). Food intake was measured to the nearest 0.1 g of the leftover food on a daily basis. The weight of organs and body was monitored.

### Survival Assay

Survival studies of leukemia‐bearing mice were performed at both Fudan University and the Institute of Hematology, Chinese Academy of Medical Sciences, according to the ethical permits in which the humane endpoint was the criterium to euthanize each mouse. Body condition score BCS‐1 (moribund state) was not exceeded in any of the experiments.

### Drug Treatment

For β3‐adrenergic receptor agonists treatment, mice were i.p. administrated with or without CL‐316243 (Cat. No. 1499, TOCRIS) at 2.5 mg kg^−1^ per day, or orally administrated with or without mirabegron (Cat. No. HY‐14773, MedChemExpress) at 10 mg kg^−1^ per day. For low‐dose chemotherapy, mice were treated with i.v. administrated with doxorubicin hydrochloride (Cat. No. MB1087, Meilunbio, China) for 3 days and i.v. administrated with cytarabine hydrochloride (Cat. No. MB5033, Meilunbio, China) for 5 days. Clinically relevant chemotherapy was twice the dose of the low‐dose chemotherapy.

### Histology and Immunofluorescence Staining

For histological analysis, femur bone, spleen, and liver were fixed with 4% paraformaldehyde (PFA) (Cat. No. MA0192, Meilunbio, China) for 12 h at room temperature. For bone tissue, decalcification was performed for ≈3 weeks before further processing. Paraffin‐embedded tissues were stained as previously described.^[^ ^31^ ^]^ Samples were sectioned into the thickness of 5 µm, mounted onto glass slides, baked at 60 °C for 1 h, deparaffinized in xylene (Cat. No. 10023418, Sinopharm chemical reagent Co., Ltd, China), and rehydrated sequentially in 99%, 95%, and 70% ethanol (Cat. No. 10009218, Sinopharm chemical reagent Co., Ltd, China). Tissue slides were counterstained with hematoxylin (Mayer's) (Cat. No. MB9897, Meilunbio, China) and eosin (Cat. No. MA0164, Meilunbio, China) before dehydration with 95% and 99% ethanol, and were then mounted with neutral balsam (Cat. No. 1004160, Sinopharm chemical reagent Co., Ltd, China). Images were photographed by a NanoZoomer Digital slide scanner (Cat. No. C13220‐01, HMAMATSU). For immunofluorescence staining, tissue slides were blocked and stained with primary antibodies including a chicken anti‐GFP antibody (Cat. No. ab13970, Abcam, 1:200), a rabbit anti‐Ki‐67 antibody (Cat. No. 9129, Cell Signaling Technology, 1:100), a rabbit anti‐cleaved caspase 3 antibody (Cat. No. 9664, Cell Signaling Technology, 1:50), a rabbit anti‐ UCP1 antibody (Cat. No. 23673‐1‐AP, Proteintech Group, 1:500), and a rabbit anti‐Perilipin‐1 antibody (Cat. No. 9349, Cell Signaling Technology, 1:1600). After rinsing, tissue samples were further stained for 45 min with secondary antibodies including anti‐chicken Alexa Fluor 488 IgG (H+L) antibody (Cat. No. ab150169, Abcam, 1:200), and anti‐rabbit Alexa Fluor 594 IgG (H+L) antibody (Cat. No. A21207, Invitrogen, 1:600). Slides were counterstained with DAPI (Cat. No. C0060, Solarbio, China). After rinsing with PBS, slides were mounted with mounting medium Fluoromount‐G (Cat. No. 0100–01, SouthernBiotech, China). Images were captured using a slide scanner system (Cat. No. Pannoramic MIDI, 3DHISTECH, Hungary), and were further analyzed using the Adobe Photoshop CS software.

### Flow Cytometry and Fluorescence‐Activated Cell Sorting (FACS)

PB samples were collected, transferred to an anticoagulation tube, and centrifugated at 1500 rpm for 5 min. Cells were resuspended in RBC lysis buffer (Cat. No. MA0207, Meilunbio, China) for 5 min to remove RBCs. For spleen or liver tissue samples, tissues were minced using a tissue grinder (Cat. No. JXFSTPRP‐CL‐BSC, Jingxin, China) at 35 Hz for 30 s for 6–8 times. Cells were then resuspended in 2% FBS‐PBS supplemented with 0.02% ethylenediamine tetraacetic acid (EDTA, Cat. No. 10009717, Sinopharm chemical reagent Co., Ltd, China). The cell suspension was filtered through a 70 µm cell strainer (Cat. No. 22363548, Fisherbrand), and RBCs were lyzed. For BM tissues, BM cells were resuspended, filtered, and RBC removed. All samples were kept in 1% PFA for detection of GFP^+^ signal by flow cytometry (CytoFLEX S, Beckman Coulter; or FACSCanto II, BD Bioscience). For human leukemia xenografts, cell suspensions were incubated with FITC‐anti‐mouse CD45 antibody (Cat. No. 103108, Biolegend, 1:100), APC‐anti‐human CD45 (Cat. No. 304037, Biolegend, 1:100) at 4 °C for 30 min in the dark. After rinsing, cells were filtered to remove cell aggregates, and 1 µg mL^−1^ DAPI (Cat. No. 422801, Biolegend) was added. Human CD45^+^ cells were detected by flow cytometry (Cat. No. FACS canto II, BD Bioscience). For cell sorting, cells were harvested from the spleen as described above. After single‐cell suspensions were made, cells were added with 1 µg mL^−1^ DAPI (Cat. No. 422801, Biolegend) to remove dead cells in flow cytometry. Samples were projected to a FACS system (Cat. No. FACSAria III, BD Bioscience), and GFP^+^ live cells were collected for immediate transplantation. Flowjo software (version 10, BD Bioscience) was used to analyze the results.

### RNA Extraction and Quantitative Real‐Time PCR

Total RNA was extracted from leukemia‐infiltrated spleen and adipose tissue using an RNAsimple Total RNA kit (Cat. No. DP419, TIANGEN). Total RNA from each sample was reversely transcribed using a Hifair II 1st Strand cDNA Synthesis SuperMix (Cat. No. 11123ES60, YEASEN, China). Reverse transcription was performed at 42 °C for 15 min, and subsequently 80 °C for 5 min to inactivate the enzyme activity. The cDNA samples were subjected to qPCR using a StepOnePlus Real‐Time PCR System (Applied Biosystems). Each sample was triplicated and in a 10 µL reaction containing Hieff qPCR SYBR Green Master Mix (Cat. No. 11203ES03, YEASEN, China), 50 nmol forward and reverse primers, and 2 µL cDNA. The qPCR protocol was executed for 60 cycles and each cycle consisted of denaturation at 95 °C for 15 s, annealing at 60 °C for 1 min, and extension at 72 °C for 1 min. The primer pairs specific for various genes used in the experiments included: mouse *Glut1* forward: 5′‐ACCAAAAGCAACGGAGAAGAG‐3′; mouse *Glut1* reverse: 5′‐GGCATTCCGAAACAGGTAACTC‐3′; mouse *Glut4* forward: 5′‐GTGACTGGAACACTGGTCCTA‐3′; mouse *Glut4* reverse: 5′‐CCAGCCACGTTGCATTGTAG‐3′; mouse *Bpgm* forward: 5′‐GGACCAGAAACTTAACAACGACG‐3′; mouse *Bpgm* reverse: 5′‐CAGGCTGTGTGAATGGACCT‐3′; mouse *Pgam2* forward: 5′‐TGGAACCAAGAGAACCGTTTC‐3′; mouse *Pgam2* reverse: 5′‐TGGCATCTTTGATAGCGGTGG‐3′; mouse *Gck1* forward: 5′‐TGAGCCGGATGCAGAAGGA‐3′; mouse *Gck1* reverse: 5′‐GCAACATCTTTACACTGGCCT‐3′; mouse *Hk2* forward: 5′‐TGATCGCCTGCTTATTCACGG‐3′; mouse *Hk2* reverse: 5′‐AACCGCCTAGAAATCTCCAGA‐3′; mouse *Pgam1* forward: 5′‐AGCGACACTATGGCGGTCT‐3′; mouse *Pgam1* reverse: 5′‐TGGGACATCATAAGATCGTCTCC‐3′; mouse *Pfkp* forward: 5′‐GAAACATGAGGCGTTCTGTGT‐3′; mouse *Pfkp* reverse: 5′‐CCCGGCACATTGTTGGAGA‐3′; mouse *Pkm* forward: 5′‐GCCGCCTGGACATTGACTC‐3′; mouse *Pkm* reverse: 5′‐CCATGAGAGAAATTCAGCCGAG‐3′; mouse *Pfkm* forward: 5′‐TGTGGTCCGAGTTGGTATCTT‐3′; mouse *Pfkm* reverse: 5′‐GCACTTCCAATCACTGTGCC‐3′; mouse *Hk1* forward: 5′‐CGGAATGGGGAGCCTTTGG‐3′; mouse *Hk1* reverse: 5′‐GCCTTCCTTATCCGTTTCAATGG‐3′; mouse *Pfkl* forward: 5′‐GGAGGCGAGAACATCAAGCC‐3′; mouse *Pfkl* reverse: 5′‐CGGCCTTCCCTCGTAGTGA‐3′; mouse *Hk3* forward: 5′‐CAGGGGACCTACAGGATTGAT‐3′; mouse *Hk3* reverse: 5′‐GAGCATCTTCGTCATAGAAGGAG‐3′; mouse *Ucp1* forward: 5′‐ AGGCTTCCAGTACCATTAGGT‐3′; mouse *Ucp1* reverse: 5′‐ CTGAGTGAGGCAAAGCTGATTT‐3′; mouse *Cidea* forward: 5′‐ TGACATTCATGGGATTGCAGAC‐3′; mouse *Cidea* reverse: 5′‐ GGCCAGTTGTGATGACTAAGAC‐3′; mouse *Prdm16* forward: 5′‐ CCAAGGCAAGGGCGAAGAA‐3′; mouse *Prdm16* reverse: 5′‐ AGTCTGGTGGGATTGGAATGT‐3′; mouse *Pparg* forward: 5′‐ TCGCTGATGCACTGCCTATG‐3′; mouse *Pparg* reverse: 5′‐ GAGAGGTCCACAGAGCTGATT‐3′; mouse *Cox4* forward: 5′‐ ATTGGCAAGAGAGCCATTTCTAC‐3′; mouse *Cox4* reverse: 5′‐ CACGCCGATCAGCGTAAGT‐3′; mouse *Actb* forward: 5′‐ GGCTGTATTCCCCTCCATCG‐3′; mouse *Actb* reverse: 5′‐ CCAGTTGGTAACAATGCCATGT‐3′.

### Immunoblot

Freshly isolated tissues were minced using a low‐temperature tissue homogenizer (Cat. No. JXFSTPRP‐CL‐BSC, Jingxin, China) and were then lyzed in a RIPA lysis buffer containing proteinase and phosphatase inhibitor cocktails (Cat. No. MA0151, Meilunbio, China; Cat. No. MB2678, Meilunbio, China; 1:100). An equal amount of protein samples from each group and a standard molecular weight marker (Cat. No. AP13L052, Life‐iLab, China) were loaded on a 10% SDS‐PAGE gel (Cat. No. AP15L945, Life‐iLab, China), followed by transferring onto a polyvinylidene difluoride (PVDF) membrane (Cat. No. IPVH00010, Millipore), which was subsequently blocked with 5% skimmed milk for 2 h. Membranes were incubated overnight at 4 °C with primary antibodies diluted in a Primary Antibody Dilution Buffer (Cat. No. MB9881, Meilunbio, China). A mouse anti‐GLUT4 antibody (Cat. No. 66846‐1‐Ig, Proteintech, China; 1:1000), a rabbit anti‐GLUT1 antibody (Cat. No. 21829‐1‐AP, Proteintech, China; 1:1000), and a mouse anti‐β‐actin antibody (Cat. No. 66009‐1‐Ig, Proteintech, China; 1:5000) were used as primary antibodies. After rigorous washing with PBS containing 0.1% Tween‐20 (Cat. No. T8220, Solarbio, China), membranes were incubated at room temperature for 2 h with a goat anti‐mouse HRP‐conjugated IgG antibody (Cat. No. AS003, ABclonal, China; 1:5000) or a goat anti‐rabbit HRP‐conjugated IgG antibody (Cat. No. AS014, ABclonal, China; 1:5000). Target proteins were visualized via a super sensitive ECL luminescence reagent (Cat. No. MA0186, Meilunbio, China) with a Molecular Imager ChemiDoc XRS System (Bio‐Rad).

### Ex Vivo PET–CT Imaging

Leukemia‐bearing mice were fasted for 6 h prior to PET–CT scanning. ^18^F‐FDG with a radiochemical purity of more than 95% was produced by a cyclotron (Siemens CTI RDS Eclips ST) using the Explora FDG4 module. Ex vivo PET–CT imaging scans and image analysis were performed using an Inveon Animal PET‐CT system (Siemens Preclinical Solution). Mice were sacrificed 1 h after intravenous injection of 3.7 MBq (100 µCi) of ^18^F‐FDG, and BAT, femur bone, spleen, and liver were dissected and immediately projected for scanning. The maximal percentage‐injected dose per gram was calculated and the standardized uptake value based on body weight (SUV‐BW) of the organs was measured in a manually drawn region of interest. Inveon Acquisition Workplace software (Siemens Medical Solutions) was used for further analysis.

### Metabolomic Analysis

Metabolomic analysis was performed by high‐pressure ion chromatography (HPIC)‐multiple reaction monitoring (MRM)‐mass spectrometry (MS)/MS with the assistance of Shanghai Biotree Biotech. The extramedullary leukemia site and BAT were homogenized and metabolites were extracted with pre‐cooled MeOH/H_2_O (3/1, v/v). The solution was centrifuged at 13800 g for 15 min at 4 °C to remove debris. Supernatants were dried and resuspended in Milli‐Q water. Stock standard solutions were prepared individually to a final concentration of 10 mmol L^−1^, and a series of calibration standard solutions were prepared by stepwise dilution.

HPIC separation was performed using a Dionex HPIC System (Cat. No. ICS‐6000, Thermo Scientific), equipped with Dionex IonPac AS11‐HC (2 × 250 mm) and AG11‐HC (2 mm × 50 mm) columns. Mobile phase A was 100 mmol NaOH in water and mobile phase D was ultrapure water. The solvent (2 mmol acetic acid in methanol) was delivered via a separate pumping system and was mixed with the effluent at a flow rate of 0.15 mL min^−1^ before entering the MS. The column temperature was set at 30 °C and the autosampler temperature was set at 4 °C. The injection volume was 5 µL. A triple quadrupole mass spectrometer (Cat. No. QTRAP 6500+, AB Sciex) equipped with an electrospray ionization (ESI) interface was used for MS detection. Typical ion source parameters were: IonSpray Voltage = −4500 V, Temperature = 450 °C, Ion Source Gas 1 = 45 psi, Ion Source Gas 2 = 45 psi, Curtain Gas = 30 psi. Standard solutions of the individual analytes were analyzed and were used to optimize the MRM parameters. Several transitions showing the highest sensitivity and selectivity were selected as “quantifiers” for quantitative monitoring. The extract‐ion chromatographs were analyzed for the quantification of 56 central carbon metabolism‐associated metabolites. The correlation coefficients (R^2^) of regression fitting were greater than 0.9964 for all the analytes, indicating a satisfactory quantitative relationship between MS responses and analyte concentrations. Metabolites were projected to their corresponding pathways using KEGG (http://www.kegg.jp/kegg/pathway.html) and were visualized using ggplot2 version 3.3.5 in the R package.

### Toxicity Analysis

PB samples of mice were collected into anticoagulation tubes and were centrifuged at 6000 rpm for 10 min to collect serum. Liver and kidney toxicity was analyzed by measuring serum levels of albumin (Cat. No. OSR6102, Beckman Coulter), alanine aminotransferase (Cat. No. OSR6107, Beckman Coulter), and aspartate transaminase (Cat. No. OSR6109, Beckman Coulter), creatinine (Cat. No. OSR6178, Beckman Coulter), and uric acid (Cat. No. OSR6198, Beckman Coulter) in clinical chemistry analyzers (Cat. No. AU5800, Beckman Coulter).

### Statistical Analysis

Statistical analysis was performed using GraphPad Prism (GraphPad, USA). Statistical differences between the two groups were determined by a two‐tailed Student's *t*‐test. *p* < 0.05 was considered statistically significant, *p* < 0.01 was very significant, and *p* < 0.001 was extremely significant. Differences among multiple groups were evaluated using a one‐way ANOVA test. The sample size was indicated in the figure legends. The data is presented as means ± s.d.

## Conflict of Interest

The authors declare no conflict of interest.

## Author Contributions

R.C., T.C., and S.X. contributed equally to this work. Y.C. generated ideas for this work. R.C., T.C., and S.X. performed most experiments and analyses. X.S., M.C., S.Z., Q.R., X.N., M.R., X.Q., K.C., and S.Z. significantly contributed to experimental execution. T.C., Y.X., Y.C., and Y.Y. provided critical reagents/resources and participated in discussions. Y.Y. and Y.C. designed experiments and wrote the manuscript.

## Supporting information

Supporting Information

## Data Availability

All data of metabolomics are openly available in EMBL‐EBI’s MetaboLights at https://www.ebi.ac.uk/metabolights, reference number MTBLS10444. The data that support the findings of this study are available from the corresponding author upon reasonable request.
