# Supplementary material for: Effective Prevention and Treatment of Acute Leukemias in Mice by Activation of Thermogenic Adipose Tissues
Source: Adv Sci (Weinh). 2024 Jul 25;11(38):2402332. doi: 10.1002/advs.202402332 (PMC11481385; doi:10.1002/advs.202402332)
Supplement: Supplementary file 1 — Supporting Information [file ADVS-11-2402332-s001.pdf]

ADVANCED SCIENCE
Open Access

Supporting Information

for *Adv. Sci.*, DOI 10.1002/advs.202402332

Effective Prevention and Treatment of Acute Leukemias in Mice by Activation of Thermogenic Adipose Tissues

Ruibo Chen, Tianran Cheng, Sisi Xie, Xiaoting Sun, Mingjia Chen, Shumin Zhao, Qingyan Ruan, Xiaolei Ni, Mei Rao, Xinyi Quan, Kaiwen Chen, Shiyue Zhang, Tao Cheng, Yuanfu Xu, Yuguo Chen*, Yunlong Yang* and Yihai Cao**

Supplemental Figures and Figure Legends

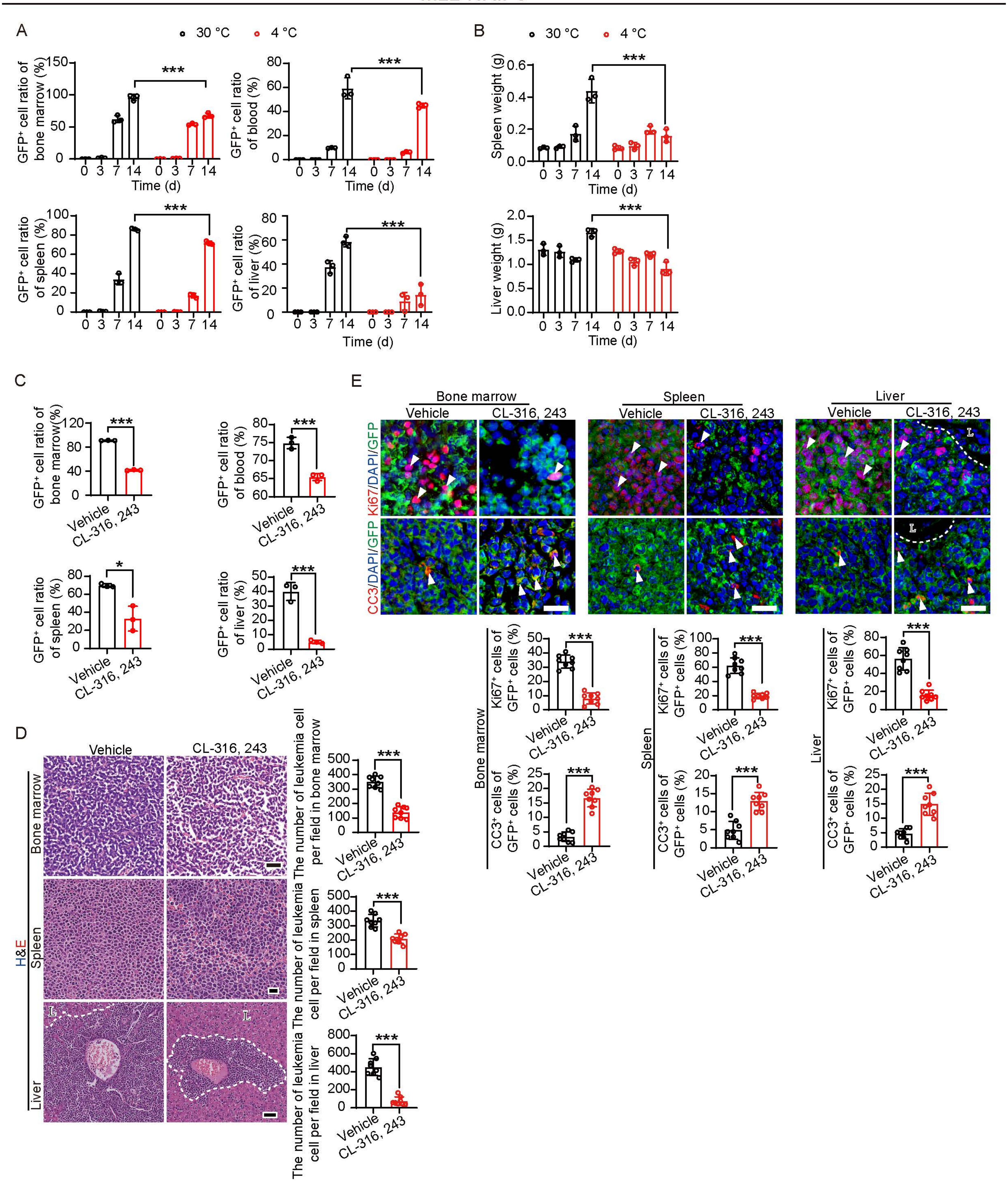
MLL-NRIP3
A
30 °C
4 °C
GFP⁺ cell ratio of bone marrow (%)
GFP⁺ cell ratio of blood (%)
GFP⁺ cell ratio of spleen (%)
GFP⁺ cell ratio of liver (%)
Time (d)
B
Spleen weight (g)
Liver weight (g)
C
Vehicle
CL-316, 243
D
H&E
Bone marrow
Spleen
Liver
The number of leukemia cell per field in bone marrow
The number of leukemia cell per field in spleen
The number of leukemia cell per field in liver
E
Ki67/DAPI/GFP
CC3/DAPI/GFP
Ki67⁺ cells of GFP⁺ cells (%)
CC3⁺ cells of GFP⁺ cells (%)

Figure S1 β3-adrenoceptor agonist suppresses MLL-NRIP3 AML progression

A. Flow cytometry analysis of GFP$^+$ cells in BM, PB, spleen, and liver in MLL-NRIP3 leukemia-bearing mice under 30 °C or 4 °C conditions for 0, 3, 7, 14 days (n = 3 mice per group). B. Weight of spleen and liver in MLL-NRIP3 leukemia-bearing mice under 30 °C or 4 °C conditions for 0, 3, 7, 14 days (n = 3 mice per group). C. After transplantation for 7 days, leukemia-bearing mice were treated with vehicle or CL-316,243 for up to 14 days. Flow cytometry analysis of GFP$^+$ cells in BM, PB, spleen, and liver in MLL-NRIP3 leukemia-bearing mice treated with vehicle or CL-316,243 (n = 3 mice per group). D. Histological analysis of BM, spleen, and liver in MLL-NRIP3 leukemia-bearing mice treated with vehicle or CL-316,243 (n = 8 random fields per group). Quantification of infiltrated leukemia cells. Scale bar in upper and middle panels, 20 μm. Scale bar in lower panel, 50 μm. E. Immunofluorescence analysis of Ki67$^+$ proliferating cells and cleaved caspase3$^+$ apoptotic cells in BM, spleen, and liver in MLL-NRIP3 leukemia-bearing mice treated with vehicle or CL-316,243 (n = 8 random fields per group). Scale bar, 20 μm. L, liver. Dashed line indicates GFP$^+$ leukemia cells. Arrowhead indicates positive signals. *$p<0.05$; **$p<0.01$; ***$p<0.001$. NS = not significant. Data presented as mean ± s.d..

Fig S2

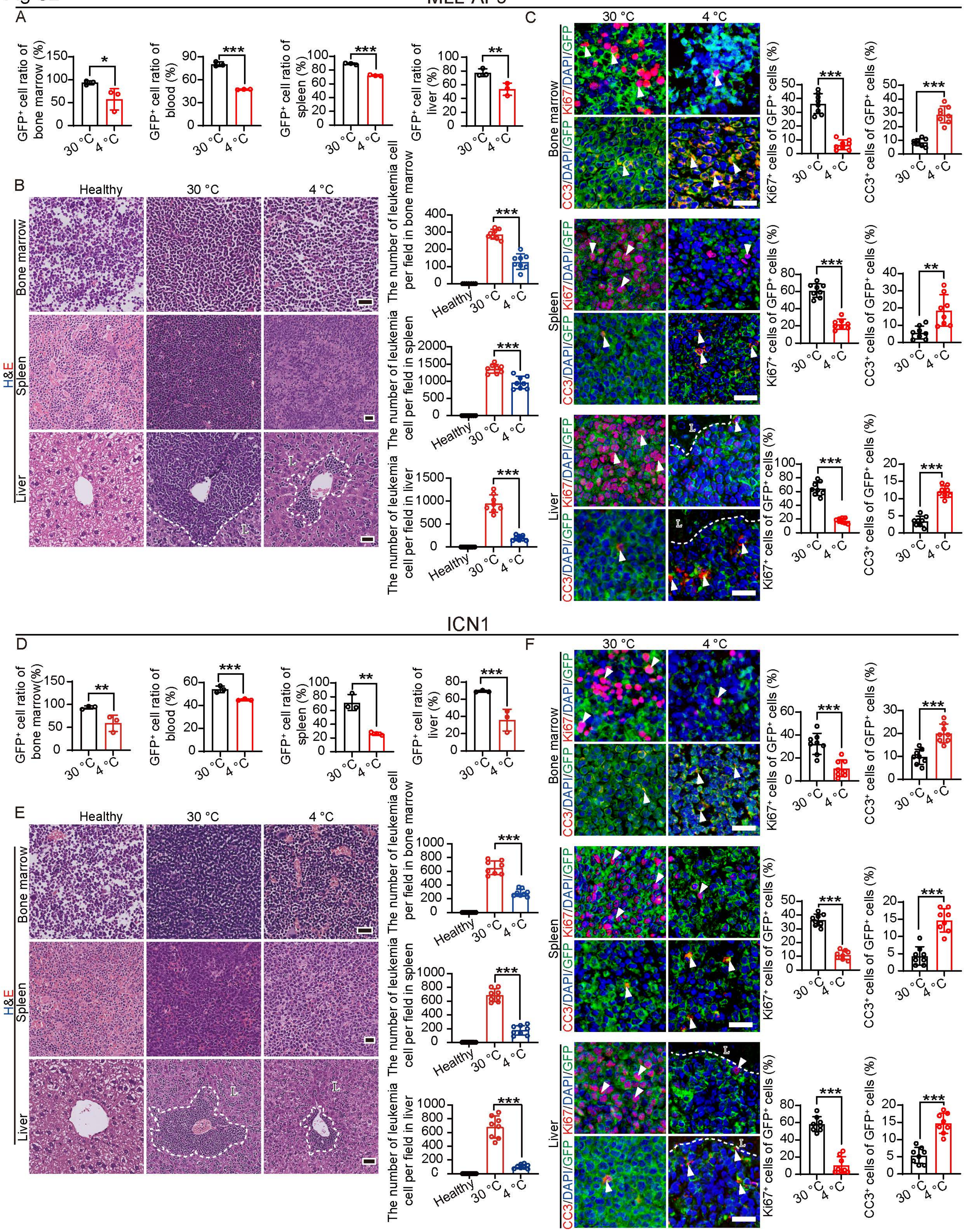

MLL-AF9
A
GFP⁺ cell ratio of bone marrow (%)
GFP⁺ cell ratio of blood (%)
GFP⁺ cell ratio of spleen (%)
GFP⁺ cell ratio of liver (%)
30 °C
4 °C
B
Healthy
30 °C
4 °C
H&E
Bone marrow
Spleen
Liver
The number of leukemia cell per field in bone marrow
The number of leukemia cell per field in spleen
The number of leukemia cell per field in liver
C
Ki67/DAPI/GFP
CC3/DAPI/GFP
Ki67⁺ cells of GFP⁺ cells (%)
CC3⁺ cells of GFP⁺ cells (%)
ICN1
D
GFP⁺ cell ratio of bone marrow(%)
E
F

Figure S2 Cold exposure suppresses the progression of MLL-AF9 AML and ICN1 ALL

A. Flow cytometry analysis of GFP^+ cells in BM, PB, spleen, and liver in MLL-AF9 leukemia-bearing mice under 30 °C or 4 °C conditions (n = 3 mice per group). B. Histological analysis of BM, spleen, and liver in MLL-AF9 leukemia-bearing mice under 30 °C or 4 °C conditions (n = 8 random fields per group). Quantification of infiltrated leukemia cells. Scale bar in upper and middle panels, 20 μm. Scale bar in lower panel, 50 μm. C. Immunofluorescence analysis of $Ki67^+$ proliferating cells and cleaved $caspase3^+$ apoptotic cells in BM, spleen, and liver in MLL-AF9 leukemia-bearing mice under 30 °C or 4 °C conditions (n = 8 random fields per group). Scale bar, 20 μm. L, liver. Dashed line indicates GFP^+ leukemia cells. Arrowhead indicates positive signals. D. Flow cytometry analysis of GFP^+ cells in BM, PB, spleen, and liver in ICN1 leukemia-bearing mice under 30 °C or 4 °C conditions (n = 3 mice per group). E. Histological analysis of BM, spleen, and liver in ICN1 leukemia-bearing mice under 30 °C or 4 °C conditions (n = 8 random fields per group). Quantification of infiltrated leukemia cells. Scale bar in upper and middle panels, 20 μm. Scale bar in lower panel, 50 μm. F. Immunofluorescence analysis of $Ki67^+$ proliferating cells and cleaved $caspase3^+$ apoptotic cells in BM, spleen, and liver in ICN1 leukemia-bearing mice under 30 °C or 4 °C conditions (n = 8 random fields per group). Scale bar, 20 μm. L, liver. Dashed line indicates GFP^+ leukemia cells. Arrowhead indicates positive signals. *$p<0.05$; **$p<0.01$; ***$p<0.001$. NS = not significant. Data presented as mean ± s.d..

Fig S3

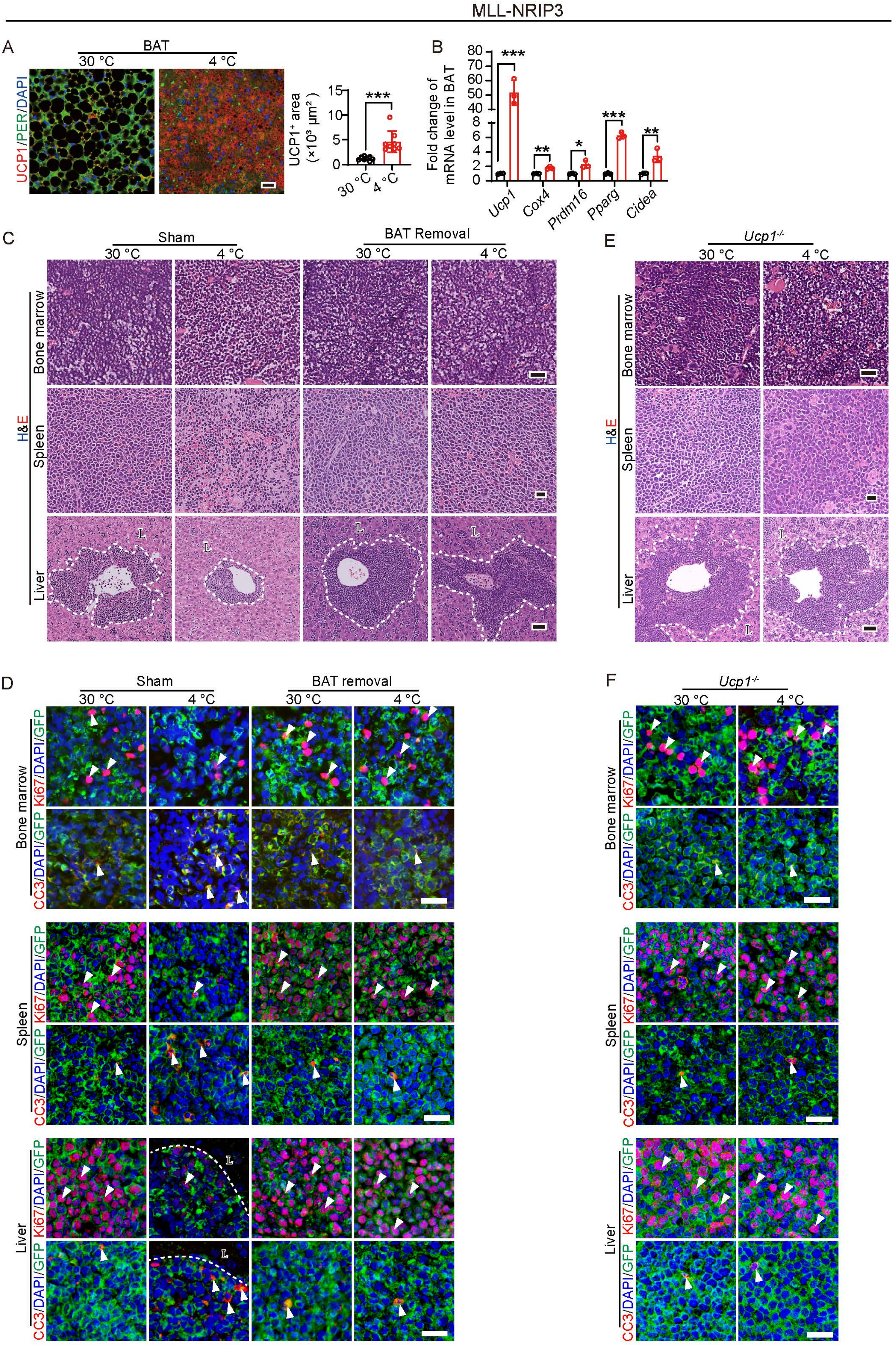

MLL-NRIP3
A
BAT
30 °C
4 °C
UCP1/PER/DAPI
UCP1⁺ area (×10³ μm²)
15
10
5
0
***
30 °C
4 °C
B
Fold change of mRNA level in BAT
80
70
60
50
40
8
6
4
2
0
***
**
*
***
**
Ucp1
Cox4
Prdm16
Pparg
Cidea
C
Sham
BAT Removal
30 °C
4 °C
30 °C
4 °C
H&E
Bone marrow
Spleen
Liver
L
E
Ucp1-/-
30 °C
4 °C
H&E
Bone marrow
Spleen
Liver
L
D
Sham
BAT removal
30 °C
4 °C
30 °C
4 °C
Bone marrow
Spleen
Liver
Ki67/DAPI/GFP
CC3/DAPI/GFP
L
F
Ucp1-/-
30 °C
4 °C
Bone marrow
Spleen
Liver
Ki67/DAPI/GFP
CC3/DAPI/GFP

Figure S3 BAT removal or *Ucp1* deletion abolishes cold-inhibited MLL-NRIP3 AML.

A. Immunofluorescence analysis of $UCP1^+$ signals in BAT in MLL-NRIP3 leukemia-bearing mice under 30 °C or 4 °C conditions (n = 8 random fields per group). Scale bar, 20 μm. B. Quantitative PCR analysis of *Ucp1*, *Cox4*, *Prdm16*, *Pparg*, *Cidea* genes in BAT in MLL-NRIP3 leukemia-bearing mice under 30 °C or 4 °C conditions (n = 3 mice per group). C. Histological analysis of BM, spleen, and liver in sham-operated or BAT-removed MLL-NRIP3 leukemia-bearing mice under 30 °C or 4 °C conditions. Scale bar in upper and middle panels, 20 μm. Scale bar in lower panel, 50 μm. D. Immunofluorescence analysis of $Ki67^+$ proliferating cells and cleaved $caspase3^+$ apoptotic cells in BM, spleen, and liver in sham-operated or BAT-removed MLL-NRIP3 leukemia-bearing mice under 30 °C or 4 °C conditions. Scale bar, 20 μm. Arrowhead indicates positive signals. L, liver. Dashed line indicates GFP^+ leukemia cells. E. Histological analysis of BM, spleen, and liver in MLL-NRIP3 leukemia-bearing $Ucp1^{-/-}$ mice under 30 °C or 4 °C conditions. Scale bar in upper and middle panels, 20 μm. Scale bar in lower panel, 50 μm. F. Immunofluorescence analysis of $Ki67^+$ proliferating cells and cleaved $caspase3^+$ apoptotic cells in BM, spleen, and liver in MLL-NRIP3 leukemia-bearing $Ucp1^{-/-}$ mice under 30 °C or 4 °C conditions. Scale bar, 20 μm. Arrowhead indicates positive signals.

Fig S4

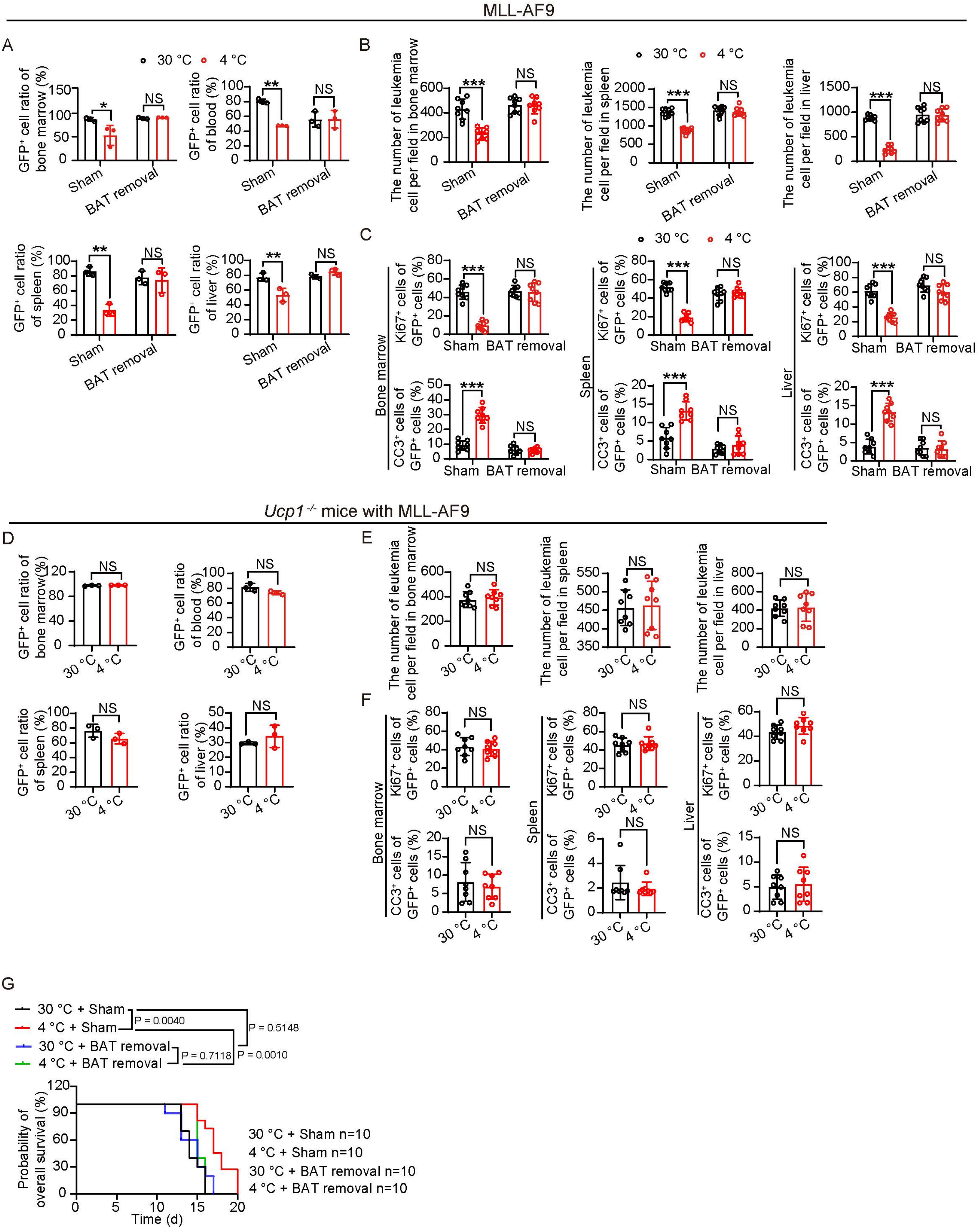

MLL-AF9
A
30 °C
4 °C
GFP⁺ cell ratio of bone marrow (%)
GFP⁺ cell ratio of blood (%)
GFP⁺ cell ratio of spleen (%)
GFP⁺ cell ratio of liver (%)
Sham
BAT removal
NS
B
The number of leukemia cell per field in bone marrow
The number of leukemia cell per field in spleen
The number of leukemia cell per field in liver
C
Bone marrow
Spleen
Liver
Ki67⁺ cells of GFP⁺ cells (%)
CC3⁺ cells of GFP⁺ cells (%)
Ucp1 -/- mice with MLL-AF9
D
E
F
G
30 °C + Sham
4 °C + Sham
30 °C + BAT removal
4 °C + BAT removal
P = 0.0040
P = 0.5148
P = 0.7118
P = 0.0010
Probability of overall survival (%)
Time (d)
30 °C + Sham n=10
4 °C + Sham n=10
30 °C + BAT removal n=10
4 °C + BAT removal n=10

Figure S4 BAT removal or *Ucp1* deletion abolishes cold-inhibited MLL-AF9 AML. A. Flow cytometry analysis of GFP^+ cells in BM, PB, spleen, and liver in sham-operated or BAT-removed MLL-AF9 leukemia-bearing mice under 30 °C or 4 °C conditions (n = 3 mice per group). B. Quantification of infiltrated leukemia cells in BM, spleen, and liver in sham-operated or BAT-removed MLL-AF9 leukemia-bearing mice under 30 °C or 4 °C conditions (n = 8 random fields per group). C. Quantification of $Ki67^+$ proliferating cells and cleaved $caspase3^+$ apoptotic cells in BM, spleen, and liver in sham-operated or BAT-removed MLL-AF9 leukemia-bearing mice under 30 °C or 4 °C conditions (n = 8 random fields per group). D. Flow cytometry analysis of GFP^+ cells in BM, PB, spleen, and liver in MLL-AF9 leukemia-bearing *Ucp1*$^{-/-}$ mice under 30 °C or 4 °C conditions (n = 3 mice per group). E. Quantification of infiltrated leukemia cells in BM, spleen, and liver in MLL-AF9 leukemia-bearing *Ucp1*$^{-/-}$ mice under 30 °C or 4 °C conditions (n = 8 random fields per group). F. Quantification of $Ki67^+$ proliferating cells and cleaved $caspase3^+$ apoptotic cells in BM, spleen, and liver in MLL-AF9 leukemia-bearing *Ucp1*$^{-/-}$ mice under 30 °C or 4 °C conditions (n = 8 random fields per group). G. Overall survival of sham-operated or BAT-removed MLL-AF9 leukemia-bearing mice under 30 °C or 4 °C conditions (n = 10 mice per group). *p<0.05; **p<0.01; ***p<0.001. NS = not significant. Data presented as mean ± s.d..

Fig S5

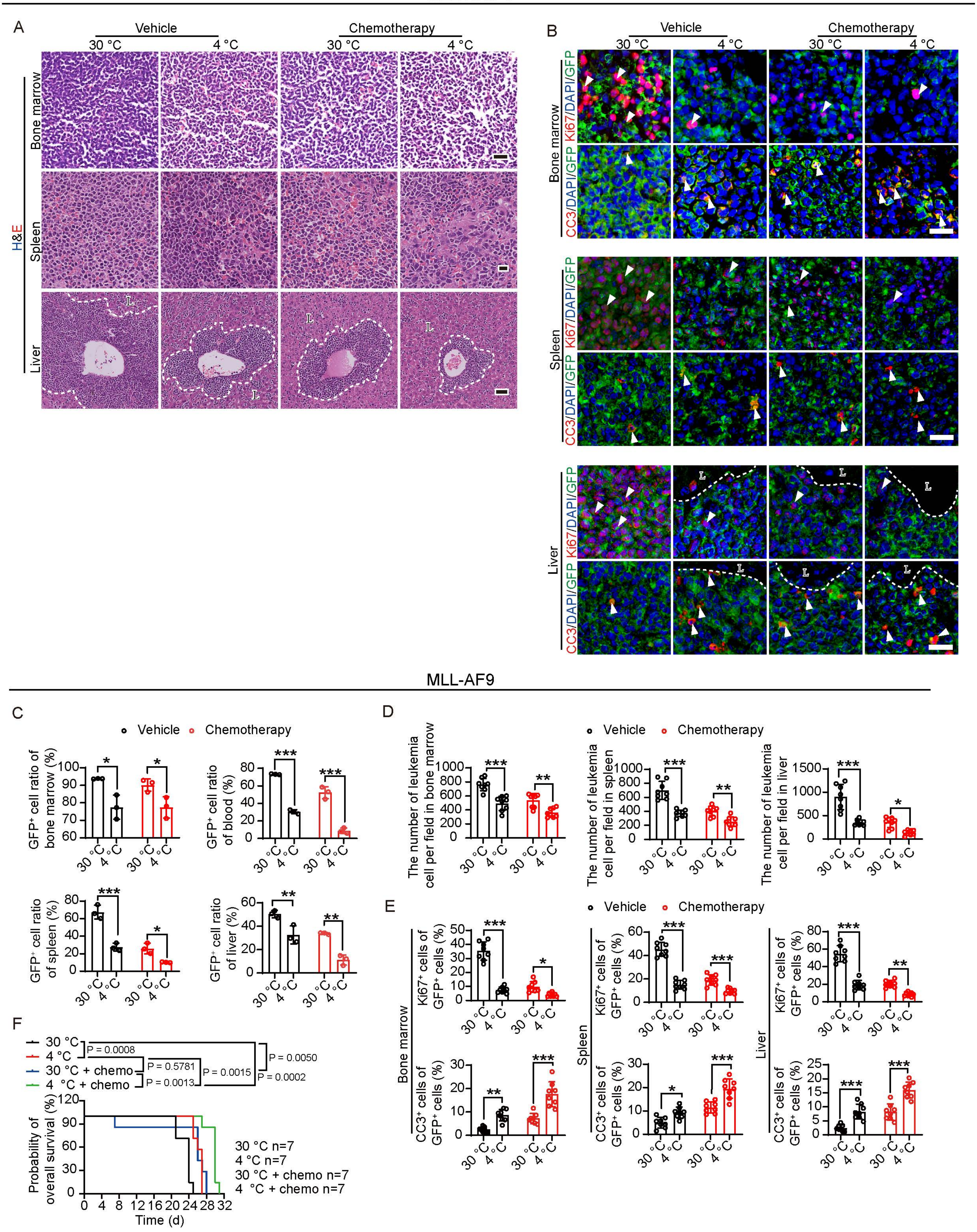
MLL-NRIP3
A
Vehicle
Chemotherapy
30 °C
4 °C
H&E
Bone marrow
Spleen
Liver
L
B
Ki67/DAPI/GFP
CC3/DAPI/GFP
MLL-AF9
C
Vehicle
Chemotherapy
GFP⁺ cell ratio of bone marrow (%)
GFP⁺ cell ratio of blood (%)
GFP⁺ cell ratio of spleen (%)
GFP⁺ cell ratio of liver (%)
D
The number of leukemia cell per field in bone marrow
The number of leukemia cell per field in spleen
The number of leukemia cell per field in liver
E
Ki67⁺ cells of GFP⁺ cells (%)
CC3⁺ cells of GFP⁺ cells (%)
F
30 °C
4 °C
30 °C + chemo
4 °C + chemo
P = 0.0008
P = 0.5781
P = 0.0013
P = 0.0015
P = 0.0050
P = 0.0002
Probability of overall survival (%)
Time (d)
30 °C n=7
4 °C n=7
30 °C + chemo n=7
4 °C + chemo n=7

Figure S5 Combination of cold exposure and chemotherapeutics synergistically suppress AML progression

A. Histological analysis of BM, spleen, and liver in vehicle- or chemo-treated MLL-NRIP3 leukemia-bearing mice under 30 °C or 4 °C conditions. Scale bar in upper and middle panels, 20 μm. Scale bar in lower panel, 50 μm. B. Immunofluorescence analysis of Ki67$^+$ proliferating cells and cleaved caspase3$^+$ apoptotic cells in BM, spleen, and liver in vehicle- or chemo-treated MLL-NRIP3 leukemia-bearing mice under 30 °C or 4 °C conditions. Scale bar, 20 μm. Arrowhead indicates positive signals. L, liver. Dashed line indicates GFP$^+$ leukemia cells. C. Flow cytometry analysis of GFP$^+$ cells in BM, PB, spleen, and liver in vehicle- or chemo-treated MLL-AF9 leukemia-bearing mice under 30 °C or 4 °C conditions (n = 3 mice per group). D. Quantification of infiltrated leukemia cells in BM, spleen, and liver in vehicle- or chemo-treated MLL-AF9 leukemia-bearing mice under 30 °C or 4 °C conditions (n = 8 random fields per group). E. Quantification of Ki67$^+$ proliferating cells and cleaved caspase3$^+$ apoptotic cells in BM, spleen, and liver in vehicle- or chemo-treated MLL-AF9 leukemia-bearing mice under 30 °C or 4 °C conditions (n = 8 random fields per group). F. Overall survival of vehicle- or chemo-treated MLL-AF9 leukemia-bearing mice under 30 °C or 4 °C conditions (n = 7 mice per group). *$p<0.05$; **$p<0.01$; ***$p<0.001$. NS = not significant. Data presented as mean ± s.d..

Fig S6

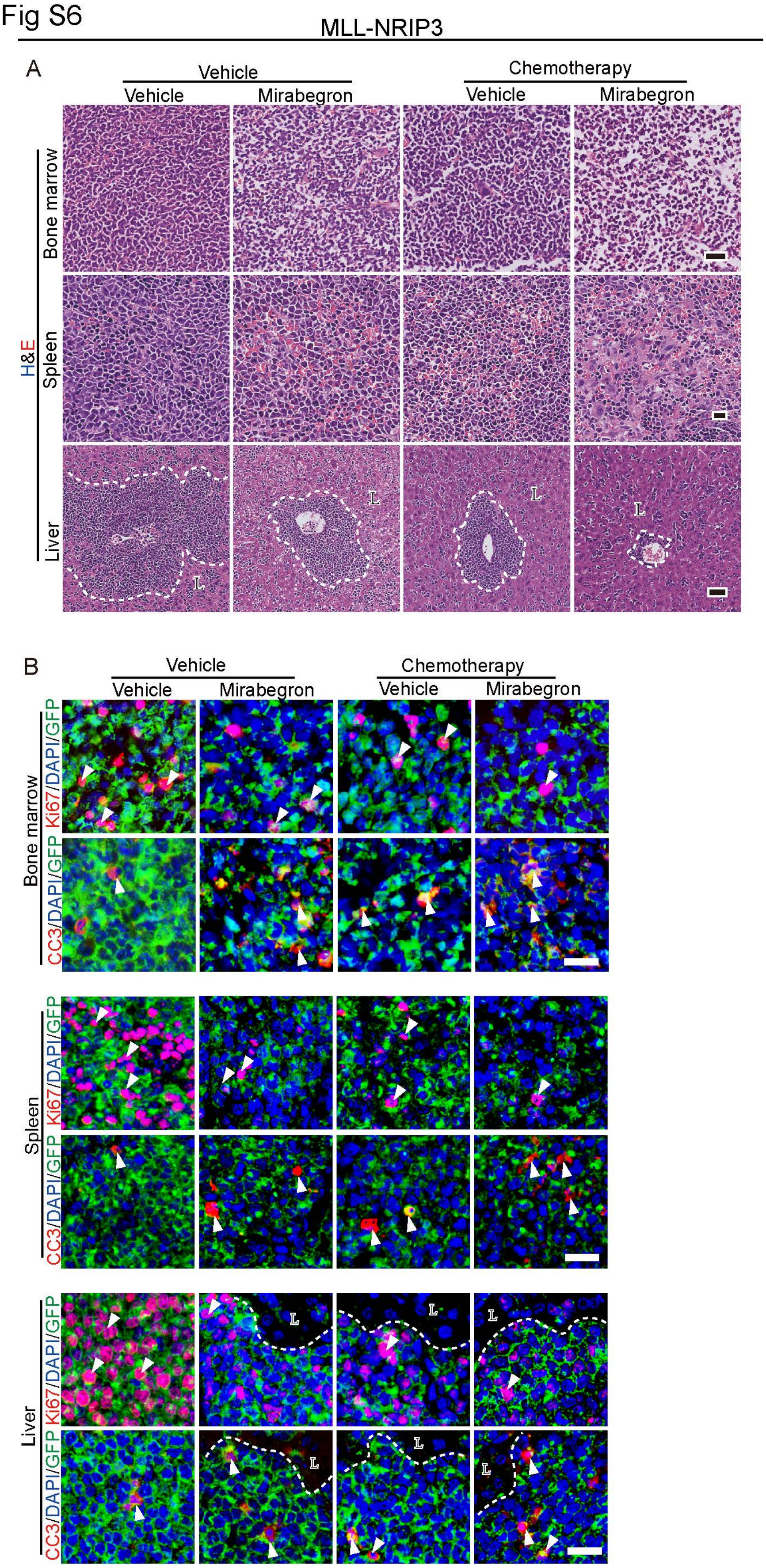

MLL-NRIP3
A
Vehicle
Chemotherapy
Vehicle
Mirabegron
Vehicle
Mirabegron
H&E
Bone marrow
Spleen
Liver
L
B
Vehicle
Chemotherapy
Vehicle
Mirabegron
Vehicle
Mirabegron
Bone marrow
Spleen
Liver
Ki67/DAPI/GFP
CC3/DAPI/GFP
L

Figure S6 Combination of mirabegron and chemotherapeutics synergistically suppress MLL-NRIP3 AML progression

A. Histological analysis of BM, spleen, and liver in vehicle- or chemo-treated MLL-NRIP3 leukemia-bearing mice under vehicle or mirabegron treatment. Scale bar in upper and middle panels, 20 μm. Scale bar in lower panel, 50 μm. B. Immunofluorescence analysis of $Ki67^+$ proliferating cells and cleaved $caspase3^+$ apoptotic cells in BM, spleen, and liver in vehicle- or chemo-treated MLL-NRIP3 leukemia-bearing mice under vehicle or mirabegron treatment. Scale bar, 20 μm. Arrowhead indicates positive signals.

Fig S7

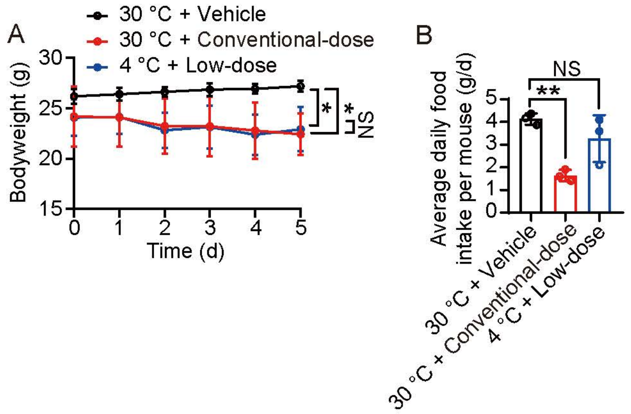

A
30 °C + Vehicle
30 °C + Conventional-dose
4 °C + Low-dose
Bodyweight (g)
30
25
20
15
0
1
2
3
4
5
Time (d)
*
*
NS
B
Average daily food intake per mouse (g/d)
5
4
3
2
1
0
NS
**
30 °C + Vehicle
30 °C + Conventional-dose
4 °C + Low-dose

Figure S7 Bodyweight and food intake of conventional chemotherapy- or combination therapy-treated mice

A. Bodyweight changes in conventional chemotherapy- or combination therapy-treated MLL-NRIP3 leukemia-bearing mice. Non-treated leukemia-bearing mice served as control (n = 3-4 mice per group). B. Food intake levels in conventional chemotherapy- or combination therapy-treated MLL-NRIP3 leukemia-bearing mice. Non-treated leukemia-bearing mice served as control (n = 3-4 mice per group). *p<0.05; **p<0.01; ***p<0.001. NS = not significant. Data presented as mean ± s.d..

Fig S8

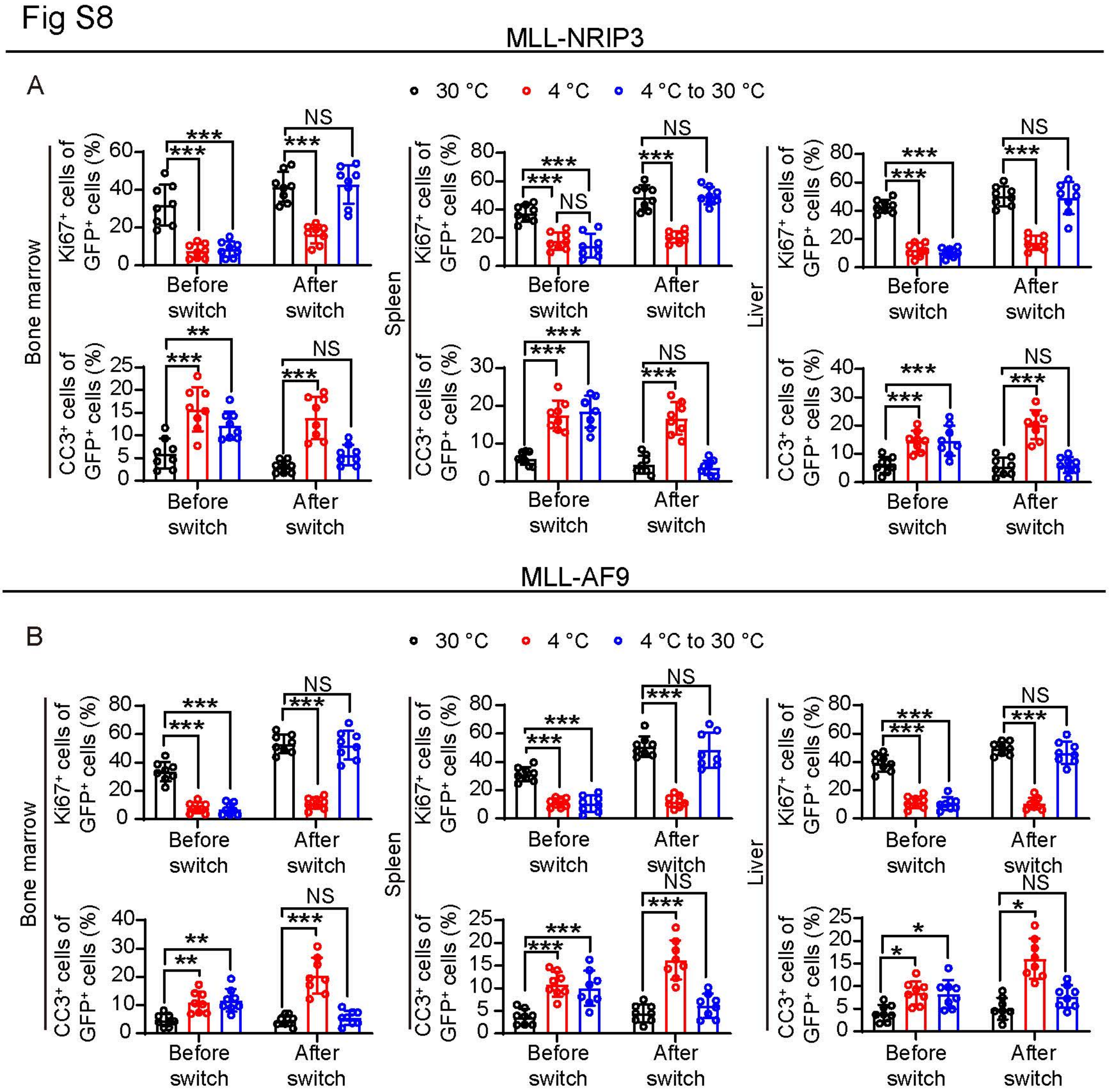

MLL-NRIP3
A
30 °C
4 °C
4 °C to 30 °C
Bone marrow
Spleen
Liver
Ki67⁺ cells of GFP⁺ cells (%)
CC3⁺ cells of GFP⁺ cells (%)
Before switch
After switch
***
**
NS
MLL-AF9
B
30 °C
4 °C
4 °C to 30 °C
Bone marrow
Spleen
Liver
Ki67⁺ cells of GFP⁺ cells (%)
CC3⁺ cells of GFP⁺ cells (%)
Before switch
After switch
***
**
*
NS

Figure S8 Proliferative and apoptotic rate of AML by continuous cold exposure

A. Quantification of $Ki67^+$ proliferating cells and cleaved $caspase3^+$ apoptotic cells in BM, spleen, and liver in 30 °C-, 4 °C-, or 4 °C-to-30 °C-exposed MLL-NRIP3 leukemia-bearing mice (n = 8 random fields per group). B. Quantification of $Ki67^+$ proliferating cells and cleaved $caspase3^+$ apoptotic cells in BM, spleen, and liver in 30 °C-, 4 °C-, or 4 °C-to-30 °C-exposed, low-dose chemotherapy-treated MLL-AF9 leukemia-bearing mice (n = 8 random fields per group). *$p<0.05$; **$p<0.01$; ***$p<0.001$. NS = not significant. Data presented as mean ± s.d..

Fig S9

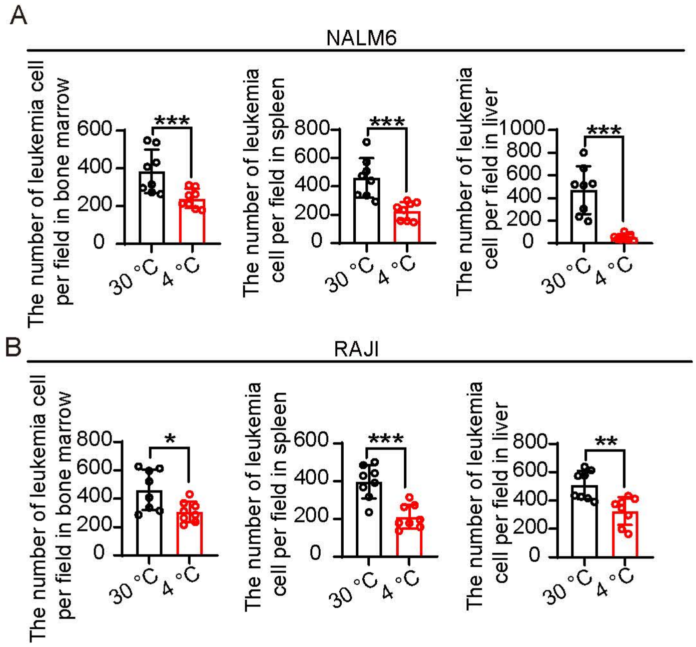
A
NALM6
The number of leukemia cell per field in bone marrow
The number of leukemia cell per field in spleen
The number of leukemia cell per field in liver
0
200
400
600
800
1000
***
30 °C
4 °C
B
RAJI
The number of leukemia cell per field in bone marrow
The number of leukemia cell per field in spleen
The number of leukemia cell per field in liver
*
***
**

Figure S9 Cold exposure suppresses leukemia progression in human leukemia xenografts

A and B. Quantification of infiltrated leukemia cells in BM, spleen, and liver in NALM6-bearing mice and RAJI-bearing mice under 30 °C or 4 °C conditions (n = 8 random fields per group). *$p<0.05$; **$p<0.01$; ***$p<0.001$. NS = not significant. Data presented as mean ± s.d..
